# A systems approach reveals species differences in hepatic stress response capacity

**DOI:** 10.1093/toxsci/kfad085

**Published:** 2023-08-30

**Authors:** Giusy Russomanno, Rowena Sison-Young, Lucia A Livoti, Hannah Coghlan, Rosalind E Jenkins, Steven J Kunnen, Ciarán P Fisher, Dennis Reddyhoff, Iain Gardner, Adeeb H Rehman, Stephen W Fenwick, Andrew R Jones, Guy Vermeil De Conchard, Gilles Simonin, Helene Bertheux, Richard J Weaver, Robert L Johnson, Michael J Liguori, Diana Clausznitzer, James L Stevens, Christopher E Goldring, Ian M Copple

**Affiliations:** Department of Pharmacology & Therapeutics, Institute of Systems, Molecular & Integrative Biology, University of Liverpool, Liverpool, L69 3GE, UK; Department of Pharmacology & Therapeutics, Institute of Systems, Molecular & Integrative Biology, University of Liverpool, Liverpool, L69 3GE, UK; Department of Pharmacology & Therapeutics, Institute of Systems, Molecular & Integrative Biology, University of Liverpool, Liverpool, L69 3GE, UK; Department of Pharmacology & Therapeutics, Institute of Systems, Molecular & Integrative Biology, University of Liverpool, Liverpool, L69 3GE, UK; Department of Pharmacology & Therapeutics, Institute of Systems, Molecular & Integrative Biology, University of Liverpool, Liverpool, L69 3GE, UK; CDSS Bioanalytical Facility, Institute of Systems, Molecular & Integrative Biology, University of Liverpool, Liverpool, L69 3GE, UK; Division of Drug Discovery and Safety, Leiden Academic Centre for Drug Research (LACDR), Leiden University, Leiden, 2311 EZ, The Netherlands; Simcyp Division, Certara UK, Sheffield, S1 2BJ, UK; Simcyp Division, Certara UK, Sheffield, S1 2BJ, UK; Simcyp Division, Certara UK, Sheffield, S1 2BJ, UK; Department of Pharmacology & Therapeutics, Institute of Systems, Molecular & Integrative Biology, University of Liverpool, Liverpool, L69 3GE, UK; Department of Hepatobiliary Surgery, Aintree University Hospital, Liverpool University Hospitals NHS Foundation Trust, Liverpool, L9 7AL, UK; Department of Hepatobiliary Surgery, Aintree University Hospital, Liverpool University Hospitals NHS Foundation Trust, Liverpool, L9 7AL, UK; Department of Biochemistry & Systems Biology, Institute of Systems, Molecular & Integrative Biology, University of Liverpool, Liverpool, L69 7ZB, UK; Translational Medicine, Non Clinical Safety, Biologie Servier, Gidy, 45520, France; Translational Medicine, Non Clinical Safety, Biologie Servier, Gidy, 45520, France; Translational Medicine, Non Clinical Safety, Biologie Servier, Gidy, 45520, France; Institut de R&D Servier Paris-Saclay, Gif sur Yvette, 91190, France; Eli Lilly and Company, Indianapolis, Indiana 46225, USA; AbbVie Inc., Pre-Clinical Safety, Chicago, Illinois 60064, USA; AbbVie Deutschland GmbH & Co. KG, DMPK-BA, Ludwigshafen, 67061, Germany; Division of Drug Discovery and Safety, Leiden Academic Centre for Drug Research (LACDR), Leiden University, Leiden, 2311 EZ, The Netherlands; Department of Pharmacology & Therapeutics, Institute of Systems, Molecular & Integrative Biology, University of Liverpool, Liverpool, L69 3GE, UK; Department of Pharmacology & Therapeutics, Institute of Systems, Molecular & Integrative Biology, University of Liverpool, Liverpool, L69 3GE, UK

**Keywords:** acetaminophen, liver injury, oxidative stress, preclinical species

## Abstract

To minimize the occurrence of unexpected toxicities in early phase preclinical studies of new drugs, it is vital to understand fundamental similarities and differences between preclinical species and humans. Species differences in sensitivity to acetaminophen (APAP) liver injury have been related to differences in the fraction of the drug that is bioactivated to the reactive metabolite N-acetyl-p-benzoquinoneimine (NAPQI). We have used physiologically based pharmacokinetic modeling to identify oral doses of APAP (300 and 1000 mg/kg in mice and rats, respectively) yielding similar hepatic burdens of NAPQI to enable the comparison of temporal liver tissue responses under conditions of equivalent chemical insult. Despite pharmacokinetic and biochemical verification of the equivalent NAPQI insult, serum biomarker and tissue histopathology analyses revealed that mice still exhibited a greater degree of liver injury than rats. Transcriptomic and proteomic analyses highlighted the stronger activation of stress response pathways (including the Nrf2 oxidative stress response and autophagy) in the livers of rats, indicative of a more robust transcriptional adaptation to the equivalent insult. Components of these pathways were also found to be expressed at a higher basal level in the livers of rats compared with both mice and humans. Our findings exemplify a systems approach to understanding differential species sensitivity to hepatotoxicity. Multiomics analysis indicated that rats possess a greater basal and adaptive capacity for hepatic stress responses than mice and humans, with important implications for species selection and human translation in the safety testing of new drug candidates associated with reactive metabolite formation.

A critical stage in the development of a new medicine is the transition from preclinical experiments to trials in humans. The success of this transition is dependent on the robust translation of preclinical findings to clinical responses, in terms of both efficacy and safety (Aithal 2010; Monticello *et al.*, 2017; Olson *et al.*, 2000). For example, there are numerous cases where a drug candidate has caused serious liver toxicity in patients despite preclinical safety assessment indicating no cause for concern (Larson *et al.*, 2005; Leslie *et al.*, 2007; Smith 2003). On the other hand, many developmental compounds deemed unsafe in preclinical species may have been found to be safe in humans. In order to minimize the occurrence of unexpected clinical toxicities and improve the efficiency of the drug development process, it is critical that decision making is informed by a high degree of confidence in preclinical safety data. Related to this, it is vital to understand fundamental similarities and differences among and between preclinical species and humans, to inform the selection of appropriate preclinical models, and so accurately predict clinical responses based on *in vivo* and *in vitro* data. Conceptually, differential adverse effects of a drug in different species may be a result of dissimilar toxicokinetics and/or toxicodynamics. More specifically, the differential response to a drug may be driven by: (a) differences in its disposition (eg, a toxic metabolite is formed to a greater extent in the sensitive species, such as with the hepatotoxin acetaminophen [APAP]; McGill *et al.*, 2012), (b) the differential expression of a target protein (eg, the equilibrative nucleoside transporter 1, in the case of fialuridine liver toxicity; Lee *et al.*, 2006), or (c) fundamental differences in the nature and/or extent of the downstream cellular response to chemical insult. Although (a) and (b) have been extensively studied in the context of various drug toxicities, (c) has garnered less attention to date.

APAP is the single largest cause of acute liver failure in the United Kingdom and United States (Bernal *et al.*, 2010; Larson *et al.*, 2005), and is commonly used as an exemplar drug for studying hepatotoxicity. APAP is metabolically bioactivated to the highly reactive N-acetyl-p-benzoquinoneimine (NAPQI) which depletes glutathione (GSH) stores, induces oxidative stress, and covalently reacts with hepatocellular proteins leading to necrosis (Ramachandran and Jaeschke 2019; Yoon *et al.*, 2016). It is well known that mice (oral LD50 approximately 350 mg/kg) are relatively sensitive to APAP liver injury compared with rats (oral LD50 approximately 2000 mg/kg) (McGill *et al.*, 2012). Previous work has indicated that this disparity is related to species differences in metabolism, with mice exhibiting a higher fraction of APAP bioactivated to NAPQI (Boobis *et al.*, 1986; Gregus *et al.*, 1988; Tee *et al.*, 1987). To account for this species difference in metabolism, and better design a comparative study of the response of mice and rats to NAPQI insult, we have used physiologically based pharmacokinetic (PBPK) modeling and simulation to identify doses of APAP yielding an equivalent hepatic burden of NAPQI in the 2 species. We combined this with a systems approach to compare changes in the hepatic transcriptome and proteome of mice and rats following exposure to equivalent NAPQI burden, and reveal marked differences between the rodent species, and humans, in the basal and adaptive capacities of hepatic stress response pathways that are known to influence APAP toxicity and other forms of drug-induced liver injury (DILI). Our findings have important implications for the selection of preclinical models during the toxicity assessment of new drug candidates.

## Materials and methods

Extended materials and methods are available in the Supplementary Data.

###  

####  

##### PBPK modeling

Physiologically based pharmacokinetic (PBPK) models were constructed using the Simcyp Animal simulator (v18r2; Certara Inc.) to predict the disposition of APAP and its metabolites arising from sulfation, glucuronidation, and cytochrome P450 mediated metabolism and glutathione conjugation of the resultant reactive-metabolite, NAPQI. The PBPK models were used to perform simulations to determine APAP oral equivalent doses (OEDs, mg/kg body weight) resulting in a total (cumulative over 24 h) hepatic NAPQI burden equivalent between the species of interest. Models were parameterized and their predictive performance verified using the observed data from the *in vivo* study reported here; exemplar simulations are provided in Supplementary Figure 1. Data from this study were then analyzed to determine the total hepatic NAPQI burden in each species based on glutathione depletion (Supplementary File 1).

##### Animals and dosing

Animal experiments were performed at Biologie Servier (France) in accordance with the European Council Directive 2010/63/EU on the protection of animals used for scientific purposes, the ARRIVE guidelines (Percie du Sert *et al.*, 2020) and the Guiding Principles in the Use of Animals in Toxicology. The studies were approved by both Biologie Servier and TransQST consortium ethical review committees. For the time course study, overnight fasted male C57Bl/6J mice (n = 5 per time point) and Sprague Dawley rats (n = 5 per time point) were administered 300 and 1000 mg/kg APAP, respectively, or vehicle (1% w/v hydroxyethylcellulose; Sigma-Aldrich) by oral gavage. At relevant time points, the animals were sacrificed by exsanguination under isoflurane anesthesia. Liver tissue and blood samples were collected at baseline (0 h) and at 3, 6, 9 and 24 h after administration of APAP or vehicle for the analyses described below. Given that the levels of serum markers of liver injury were significantly elevated in mice at the earliest 3 h time point, a separate *in vivo* study was conducted in mice administered 300 mg/kg APAP (by oral gavage) to collect samples at earlier time points (0.5, 1, 2, and 3 h, n = 5–7) to better reflect the initial increase in serum markers of liver injury.

##### Transcriptomic analysis

Total RNA (5 µg) was processed per the standard Affymetrix protocol for microarray target preparation (Affymetrix GeneChip Mouse Genome 430 2.0 Array or Affymetrix GeneChip Rat Genome 230 2.0 Array). Differential expression analyses were carried out using the limma package (v3.42.2) in R (Ritchie *et al.*, 2015). Genes were considered differentially expressed when the adjusted *p* value (Benjamini-Hochberg correction) was less than .05 and there was at least a 1.5-fold change in expression.

ClusterProfiler package (v3.18.0) (Yu *et al.*, 2012) was used for gene set enrichment analysis (GSEA). Gene ontology (GO) biological processes (Ashburner *et al.*, 2000) were considered significantly enriched with a FDR-corrected *p*_adj_ < .05 and absolute Normalized Enrichment Score (NES) >1.5. The full list of significantly enriched GO biological processes in both species at all time points is provided in Supplementary File 2.

Weighted gene coexpression network analysis (WGCNA) was performed on the differentially expressed genes (DEGs) at each time point using the DILI TXG-MAPr tool (https://txg-mapr.eu/), and an eigengene score (EGs, or module score), was calculated for each module as previously described (Sutherland *et al.*, 2018). The average absolute EGs were calculated by averaging the absolute scores across all the modules in each species at each time point. The list of the top 50 most significant genes in each module (ordered by significance) and their relative log_2_ fold-change can be found in Supplementary File 3.

##### SWATH proteomics

SWATH proteomics was performed on liver tissue lysates. A pool of 20 samples/species representative of all the experimental conditions was used to generate the reference spectral libraries for mouse and rat, which included 5253 and 5823 proteins, respectively. SWATH data from individual samples were aligned with the spectral libraries using DIA-NN (v1.8) (Demichev *et al.*, 2020) and annotated using the reference proteomes (UP000000589 and UP000002494 for mouse and rat, respectively). Normalization and differential expression analyses were carried out using the DEqMS package (v1.8.0) (Zhu *et al.*, 2020) in R. Ingenuity Pathway Analysis software (IPA; Qiagen) was used to investigate enriched canonical pathways and toxicity functions (IPA-Tox) in the 2 species. Since the mouse and rat SWATH datasets were analyzed separately, log_2_ transformed normalized protein expression values of orthologous proteins from the 0 h control animals (untreated) were ranked and grouped into 10 bins within each dataset for comparison of basal protein abundances across species (Wang *et al.*, 2022). Proteins with the lowest protein abundance values were assigned to bin 1, whereas those with the highest abundance values were assigned to bin 10. Proteins that were not detected (NA, not available) were assigned a bin value of 0. See Supplementary File 4 for more details.

##### Analysis of public RNA-seq data

To assess basal expression levels of relevant genes in liver tissue from humans, mice, and rats, publicly available RNA-seq data from Cardoso-Moreira *et al.* (2019) were used (https://apps.kaessmannlab.org/evodevoapp/). Hepatic mRNA levels (reads per kilobase per million, RPKM) in humans (ArrayExpress E-MTAB-6814), mice (E-MTAB-6798), and rats (E-MTAB-6811) were compared. Human samples were grouped as follows: “neonates,” “infants” (6–9 months), “toddlers” (2–4 years), “school” (7–9 years), “teenagers” (13–19 years), “adults” (25–32 years), “middle-aged” (45–54 years), and “seniors” (58–63 years). Mouse samples (strain CD-1, RjOrl: SWISS) were collected at week 0, 3, 14, 28, and 63, whereas rat samples (strain Holtzman Sprague Dawley) were collected at week 0, 3, 7, 14, 42, and 112.

##### Human liver tissue

Human liver tissue was obtained by qualified medical staff at Aintree University Hospital (Liverpool, UK). All patients donated tissue as part of planned liver resections (see Supplementary Table 1 for details). Written, informed consent was obtained from each patient. The study protocol was approved by the National Health Service North West-Liverpool Central Research Ethics Committee (11/NW/0327) and in accordance with both the Declarations of Helsinki and Istanbul.

##### Statistical analysis

Statistical analysis was performed using StatsDirect 3 Software. Normal distribution was assessed by the Shapiro-Wilk test. A 2-tailed Student’s unpaired *t* test or 1-way ANOVA was used if normality was indicated. For nonnormally distributed data, a 2-tailed Mann-Whitney *U* or Kruskal-Wallis test was applied. Results were considered significant if *p* ≤ .05.

## Results

###  

#### Identification of pharmacokinetically equivalent doses of APAP in mice and rats

**Figure 1.**
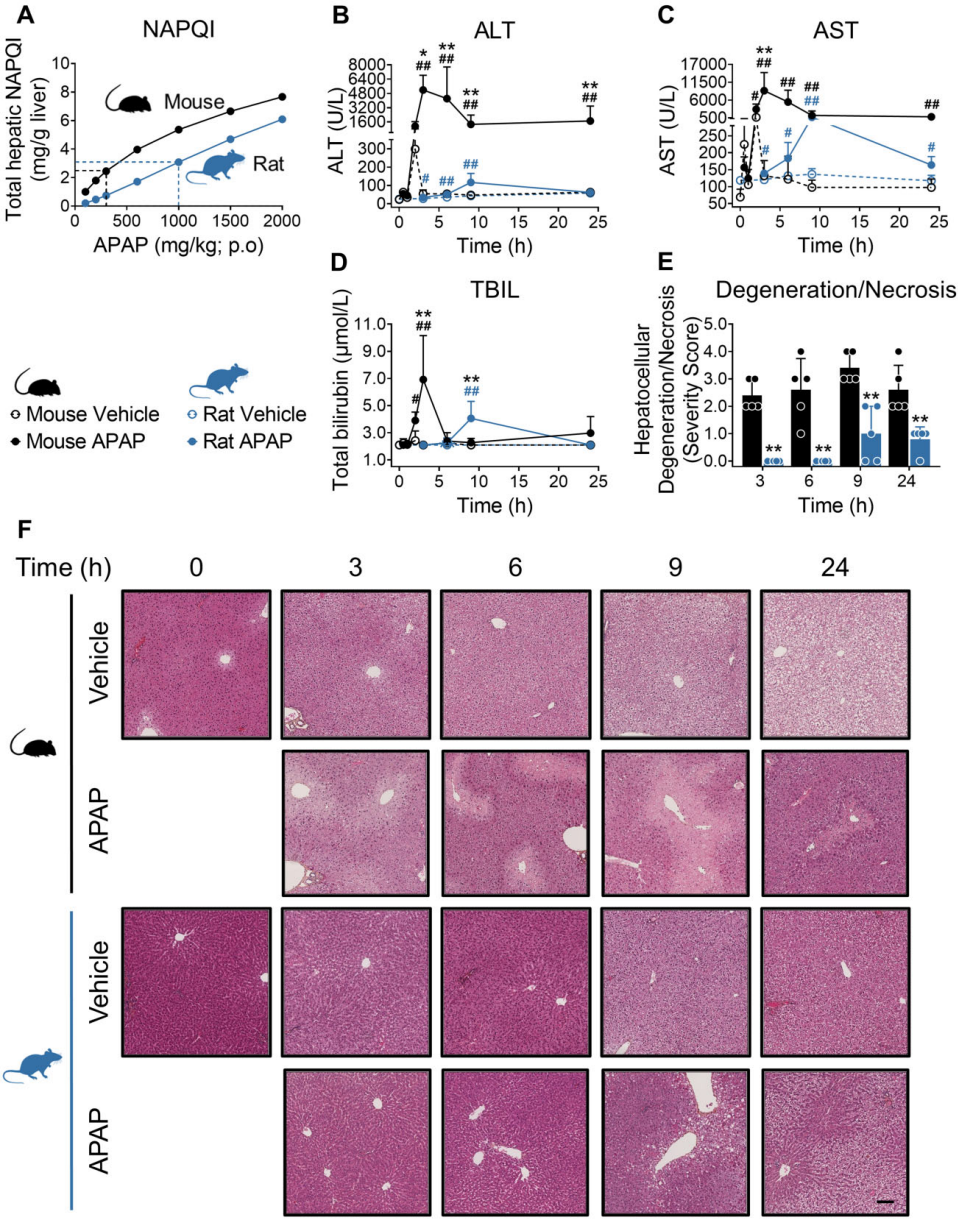
Hepatocellular injury after equivalent hepatic NAPQI burden occurs more quickly and to a greater magnitude in mice compared with rats. A, Oral equivalent doses (OEDs) of APAP derived from PBPK model simulations predicted to give similar levels of total hepatic NAPQI burden in mice (300 mg/kg) and rats (1000 mg/kg). Liver injury serum markers (ALT [B], AST [C] and total bilirubin [TBIL, D]) and hepatocellular degeneration/necrosis (E) in mice and rats exposed to 300 and 1000 mg/kg APAP, respectively. Values are mean±SD (n = 5). *p* Values are denoted as ^#^*p* < .05, and ^##^*p* < .01 (APAP-treated against time-matched control animals) or *p < .05, and **p < .01 (APAP-treated mice vs rats, Mann-Whitney *U* test). E, Representative HES images, scale bar = 100 μm.

Although modeling the impact on drug exposure of species differences in metabolism is routine, to date little has been done to rigorously compare tissue responses to a given chemical entity across preclinical species in order to identify the species that can better translate to humans. Our preliminary 24 h dose-ranging study with the model hepatotoxin APAP in male C57Bl/6J mice and Sprague Dawley rats confirmed previous reports (McGill *et al.*, 2012) that equivalent liver injury could not be achieved in the 2 species, on the basis of changes in serum biomarkers and the extent of centrilobular hepatocellular degeneration/necrosis (Supplementary Figs. 2 and 3). This study, in agreement with previous reports (Ramachandran and Jaeschke 2019), confirmed that 300 mg/kg is a nonlethal, hepatotoxic dose of APAP in mice (Supplementary Figs. 2 and 3). We therefore used PBPK modeling and simulation to identify 300 and 1000 mg/kg APAP as OEDs resulting in an equivalent hepatic NAPQI burden in mice and rats, respectively (Figure 1A). We used these doses of APAP to investigate the temporal liver tissue responses of the 2 species under conditions of equivalent NAPQI insult. In mice, 300 mg/kg APAP resulted in significant elevations of serum ALT, AST activities, and TBIL concentration that peaked 3 h after dosing (Figs. 1B–D). The response to oral administration of APAP was more rapid than previous reports (Corcoran and Wong 1986; Ito *et al.*, 2003), and likely related to enhanced absorption associated with the 1% hydroxyethylcellulose vehicle. Although significant elevations of serum ALT, AST activities, and TBIL concentration were also observed in rats dosed with 1000 mg/kg APAP, the peak of the response was detected 9 h after dosing (ie, later than in mice), and the magnitude of change relative to vehicle treated controls was muted by comparison (Figs. 1B–D). Microscopic findings correlated with these serum biomarker changes. In mice, acute degeneration of centrilobular hepatocytes was observed as early as 2 h postdose (Figs. 1E and 1F and Supplementary Figs. 4 and 5). Degeneration progressed to hepatocellular necrosis over time with peak severity observed at 9 h postdose. In rats, centrilobular hepatocyte necrosis was only observed at 9- to 24-h postdose and occurred at lower incidence and severity relative to this finding in mice (Figs. 1E and 1F and Supplementary Figs. 4 and 5). As demonstrated by serum ALT and AST activities returning toward baseline, APAP liver injury was resolving by 24 h in rats, but remained relatively higher at the same time point in mice (Figs. 1B–F). Taken together, these findings demonstrate that hepatocellular injury occurs more rapidly and to a greater extent in mice relative to rats when challenged with an equivalent hepatic NAPQI burden.

#### Confirmation of equivalent chemical insult in the livers of mice and rats

**Figure 2.**
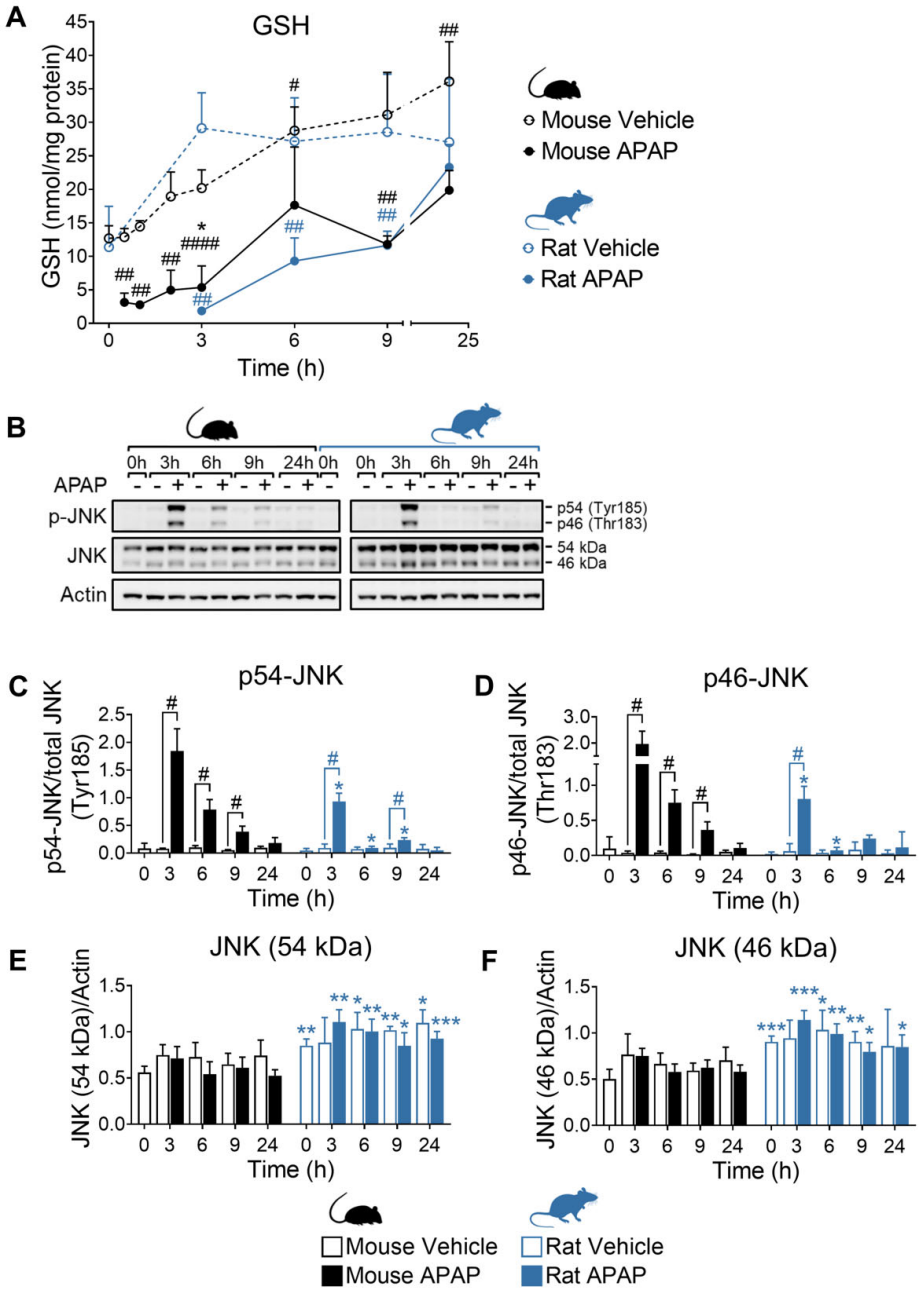
Confirmation of equivalent chemical insult in mice and rats. A, Total hepatic GSH in mice and rats exposed to 300 and 1000 mg/kg APAP respectively. Dotted lines indicate vehicle control animals. Values are mean±SD (n = 5–12). B, APAP-induced phosphorylation of JNK at Tyr185 (p56) and Thr183 (p46). Samples are pooled from 5 animals per time point. Uncropped blots are shown in Supplementary Figure 7. C–F, Densitometric analysis of immunoblots. Values are mean±SD (n = 4). Unpaired *t* test or Mann-Whitney *U* test, as appropriate. *p* Values are denoted as *p < .05, **p < .01, and ***p < .001 (mouse vs rat for each condition/time point), or ^#^*p* < .05, ^##^*p* < .01, and ^####^*p* < .0001 (APAP-treated animals vs vehicle controls).

NAPQI is inherently unstable and difficult to quantify bioanalytically. Therefore, as NAPQI is known to deplete hepatic GSH as a prerequisite for liver injury (Ramachandran and Jaeschke 2019; Yoon *et al.*, 2016), we first quantified levels of hepatic GSH in order to confirm the equivalence of chemical insult at the doses of APAP used in mice and rats. In both species, there was a time-dependent increase in GSH in vehicle-treated animals (Figure 2A), reflecting the restoration of normal resting levels following the reintroduction of food after the 16 h period of fasting prior to APAP dosing. Consistent with the metabolic bioactivation of APAP to NAPQI, there was a significant and equivalent early reduction in GSH in mice and rats treated with APAP (Figure 2A). In mice, this early depletion was followed by a time-dependent increase in GSH, which reached 55% of the level quantified in the livers of vehicle-treated controls at 24 h (Figure 2A). In rats, however, GSH was restored to 86% of the level of vehicle controls by this point (Figure 2A), indicating an enhanced capacity of the rat to adapt to the equivalent chemical insult. In addition, pharmacokinetic analysis of the plasma levels of the GSH, N-acetylcysteine, and cysteine conjugates of APAP revealed similar levels in the 2 species (Supplementary Figure 6). In keeping with the larger dose of APAP administered to rats, both the glucuronide and the sulfate conjugates were detected at higher concentrations in time-matched plasma samples compared with mice (Supplementary Figure 6).

Total hepatic NAPQI burden was calculated to assess whether PBPK modeling and simulation had indeed predicted OEDs between the 2 species. Based on the extent of GSH depletion, and assuming a 1:1 stoichiometry between NAPQI and GSH, total hepatic NAPQI burdens of 1.15 and 0.87 mg/g liver were calculated for mouse and rat, respectively (Supplementary File 1). These calculations from the study data indicate that the PBPK-predicted OEDs for mouse and rat, 300 and 1000 mg/kg, respectively, achieved total hepatic NAPQI burdens within 1.3-fold between the 2 species. Absolute PBPK predictions of total hepatic NAPQI burden were within 3.6-fold of values derived from experimental data for both species (predicted vs experimental total hepatic NAPQI; 3.19 vs 1.50 mg for mouse, 27.73 vs 7.83 mg for rat).

In a further demonstration of the comparable extent of chemical insult in the 2 species, we detected phosphorylation of c-Jun N-terminal kinase (p-JNK) in mice and rats peaking 3 h after APAP administration (Figs. 2B–D and Supplementary Figure 7). Phosphorylation and subsequent mitochondrial translocation of JNK are reported to be a critical step in the mechanism of APAP hepatotoxicity (McGill *et al.*, 2012). Although the levels of p54- and p46-JNK appeared relatively higher in mice when normalized to the total JNK level, this was largely influenced by the higher basal level of total JNK in rats (Figs. 2B, 2E, and 2F and Supplementary Figure 7), as reported by others (McGill *et al.*, 2012). Together, these data confirm the equivalence of chemical insult in the livers of mice and rats treated with 300 and 1000 mg/kg of APAP, respectively.

#### Rats exhibit a more robust activation of adaptive stress response pathways than mice in response to an equivalent NAPQI insult

**Figure 3.**
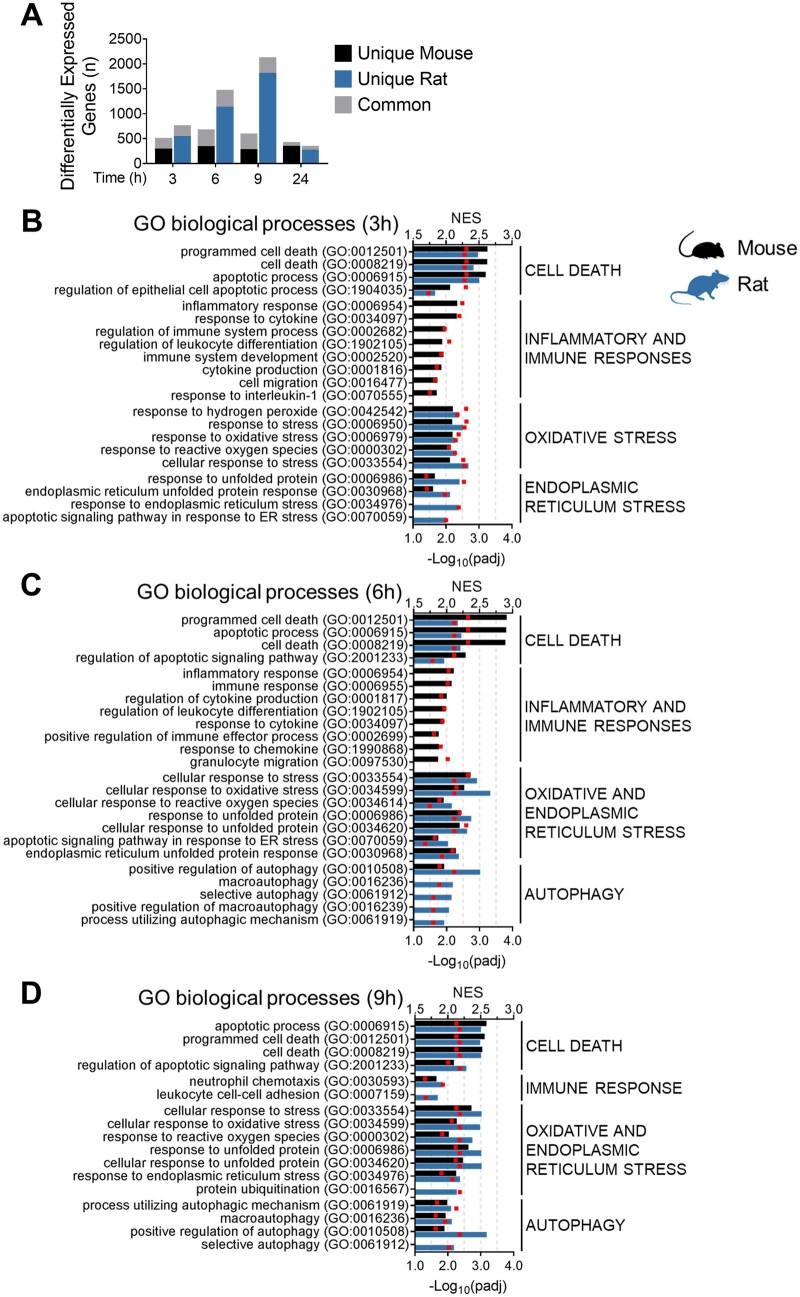
Transcriptomic analysis reveals a more robust activation of adaptive stress response pathways in the liver of APAP-treated rats, compared with mice. A, Number of genes that were uniquely responsive to APAP in mice and rat (black and blue bars, respectively; *p*_adj_ < .01, fold change >1.5 or <−1.5, comparison against time matched vehicle controls). Stacked grey bars indicate DEGs in common between the 2 species. GSEA at (B) 3, (C) 6, and (D) 9 h after APAP treatment. Bars represent normalized enrichment scores (NES) of selected GO terms for both species. Individual GO terms (*p*_adj_ < .05) were clustered into parent terms. The bar is missing for nonsignificant GO terms. Red squares represent −log_10_ of *p*_adj_.

**Figure 4.**
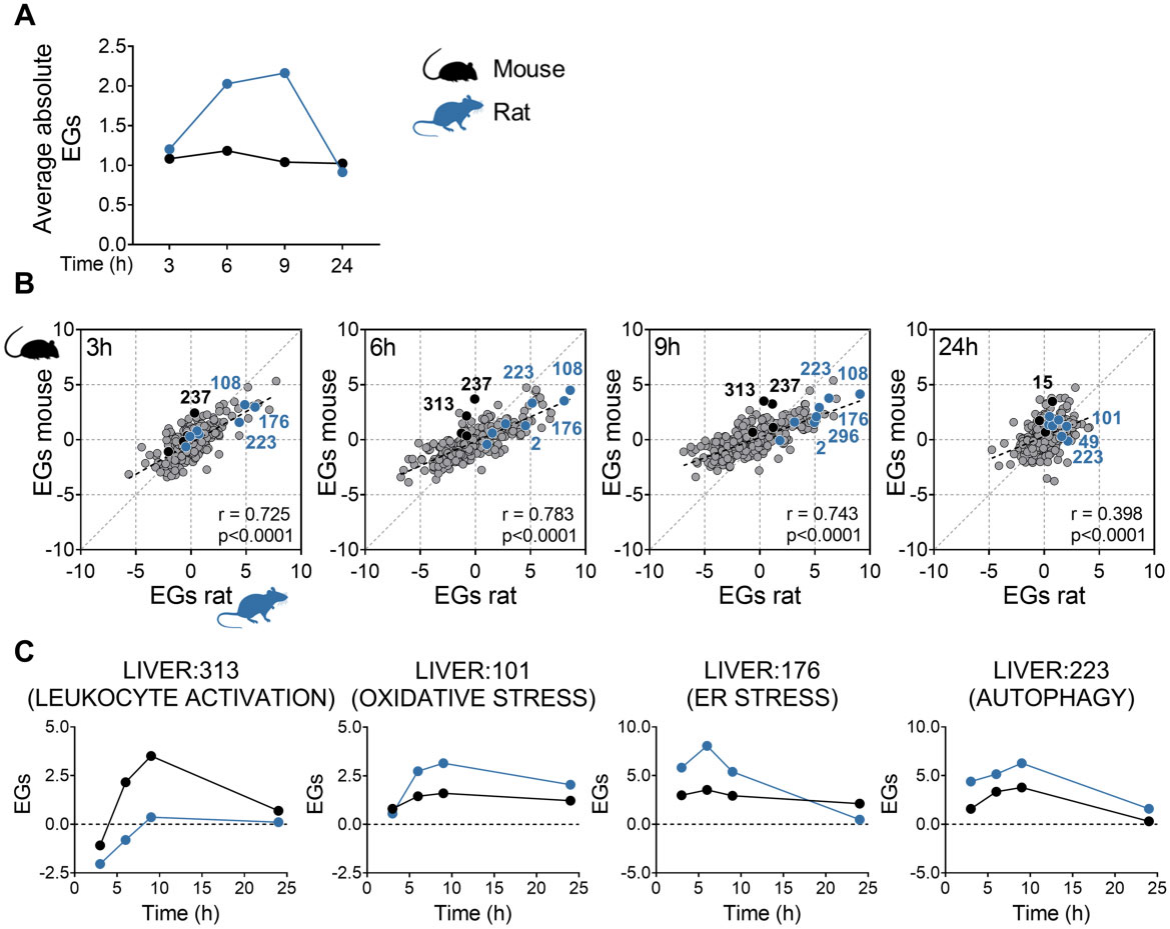
Rats show a more robust overall transcriptional response to equivalent NAPQI insult than mice. A, WGCNA average absolute EGs in APAP-treated mice and rats. B, Correlation plots of module EGs at the indicated time points. Pearson correlation coefficient (r) and *p* value for each comparison are shown. EGs for relevant modules that are higher in mouse are depicted in black (LIVER:237, negative regulation of cell proliferation; LIVER:313, leukocyte activation; LIVER:15, cell death), whereas those higher in rat are depicted in blue (LIVER:108, LIVER:101, and LIVER:49, oxidative stress response; LIVER:176, endoplasmic reticulum stress response; LIVER:223, LIVER:2, and LIVER:296, autophagy). C, EGs comparison across species for selected modules.

To explore the tissue responses of mice and rats to an equivalent NAPQI insult, we performed transcriptomic analysis on liver tissue collected from each species following administration of vehicle or the OEDs of APAP. The expression of several hundred genes was commonly altered in both mice and rats over the 24 h study period (Figure 3A), indicating a degree of similarity in the transcriptional response to APAP across the species. However, although approximately 300 genes were uniquely responsive to APAP at each time point in the mouse, a far greater number of genes (peaking at 1819 genes by 9 h post-APAP administration) were found to be differentially expressed only in the rat (Figure 3A), suggesting a more robust overall transcriptional response to the equivalent NAPQI insult in the latter species. Consistent with the relative degrees of drug-induced tissue injury observed in the 2 species, mice were found to exhibit a stronger early activation of processes involved in regulation of cell death, apoptosis, and inflammatory and immune responses, compared with rats (Figs. 3B–D). Moreover, the activation of these processes and associated genes had diminished by 24 h after APAP administration in rats but not in mice (Supplementary Figure 8), suggestive of a more robust adaptation in the former species. On the other hand, rats exhibited a stronger activation of the Nrf2-driven response to chemical and oxidative stress, along with markers of the endoplasmic reticulum stress response and autophagy, which is a catabolic process that enables the recycling of cellular components and damaged organelles under conditions of stress (Chun and Kim 2018) (Figs. 3B–D and Supplementary Figure 8 and File 2). The altered expression of representative genes from these processes was confirmed at each time point in the 2 species via qPCR (Supplementary Figure 9), which showed an excellent correlation with the corresponding transcriptomics data (Supplementary Figure 10). We also confirmed that the basal expression level of Nrf2-regulated genes was not differentially influenced by the heavy metal content of the rodent diet (Mesnage *et al.*, 2015) (Supplementary Figure 11), in light of previous reports that arsenic can modulate Nrf2 activity (Janasik *et al.*, 2018; Lau *et al.*, 2013). Our findings were further confirmed by WGCNA using the liver TXG-MAPr tool (https://txg-mapr.eu/, manuscript in preparation). Rats showed a higher average absolute eigengene score (EGs) across all modules (Figure 4A) and, although the overall module responses were well correlated between the 2 species, responses were typically stronger in rats compared with mice (Figure 4B). Indeed, rats exhibited a higher perturbation of modules associated with oxidative and endoplasmic reticulum stress responses, as well as autophagy and other adaptive processes (eg, ribosome biogenesis, which is associated with hepatocellular hypertrophy; Sinturel and Gachon 2017) when compared with mice (Figs. 4B and 4C and Supplementary Figure 12 and File 3). Together, these data indicate that the more robust activation of Nrf2 and other adaptive stress responses in rats reflects a fundamental species difference in the tissue response to the equivalent NAPQI insult.

**Figure 5.**
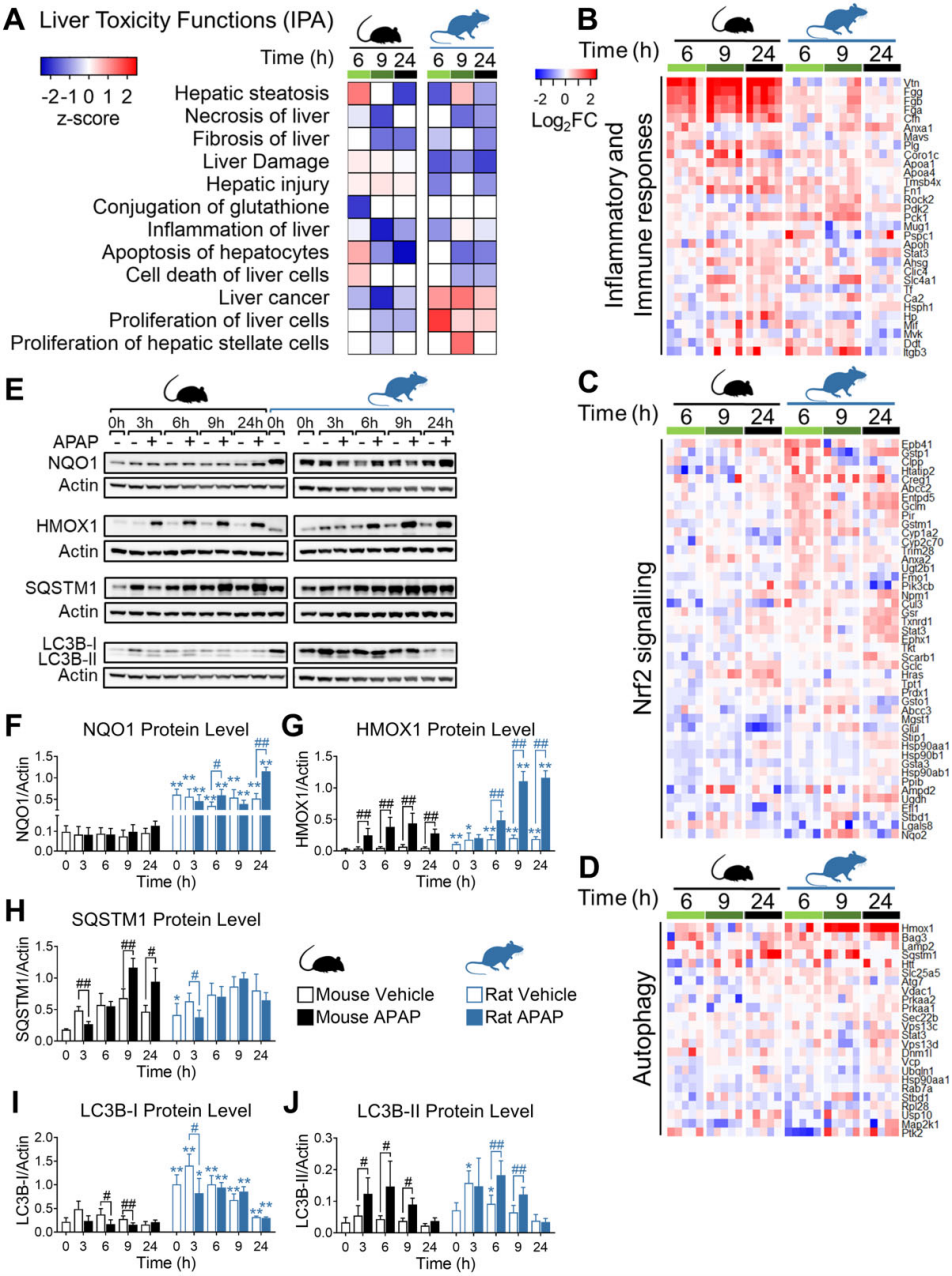
Proteomic analysis corroborates the more robust activation of adaptive stress response pathways in the liver of APAP-treated rats, compared with mice. A, Comparative toxicity analysis in the liver of APAP-treated animals (n = 5, SWATH proteomics). Heatmap plots showing changes in the expression of proteins involved in (B) inflammatory and immune responses, (C) Nrf2-signalling, and (D) autophagy (log_2_ fold-change vs time-matched vehicle control animals). E, Protein expression levels of NQO1, HMOX1, SQSTM1, LC3B-I, and -II. Samples are pooled from 5 animals per time point. Uncropped blots are shown in Supplementary Figure 7. F–J, Densitometric analysis of immunoblots. Protein levels were normalized to β-actin. Values are mean±SD (n = 5). Mann-Whitney *U* test. *p* Values are denoted as *p < .05, and **p < .01 (mouse vs rat for each condition/time point), or ^#^*p* < .05, and ^##^*p* < .01 (APAP-treated animals vs vehicle controls).

To confirm that the apparent species differences in response to the equivalent NAPQI insult were preserved at the protein level, quantitative proteomics analysis was carried out on liver tissues collected from the vehicle and APAP-treated animals. Given the time required for transcriptional responses to manifest in altered protein expression levels, we focused on the samples collected 6, 9, and 24 h after dosing, and performed IPA to understand the pathway level effects of the observed protein expression changes. Liver tissue from APAP-treated mice showed activation of toxicity functions associated with liver injury and cell apoptosis, whereas liver tissue from rats exhibited stimulation of hepatocyte proliferation processes, further supporting the different extents of adaptation between the 2 species (Figure 5A). Consistent with the observed gene level changes, liver tissue from APAP-treated rats showed a lower increase in the expression of proteins involved in inflammatory and immune responses (Figure 5B), and a greater increase in the expression of proteins involved in the Nrf2 oxidative stress response and autophagy (Figs. 5C and 5D and Supplementary Figure 13), compared with mice. The altered expression of representative proteins from these processes (NQO1, HMOX1, SQSTM1, and LC3B) was confirmed at each time point in the 2 species via immunoblotting (Figs. 5E–J and Supplementary Figure 7). Notably, in the case of LC3B, which is commonly used as a marker of autophagy (Mizushima and Yoshimori 2007), both the cytosolic LC3B-I and the autophagosome-bound LC3B-II were expressed at higher levels in the livers of rats at the 0 h time point (ie, following the 16 h fasting period) (Figs. 5I and 5J). In addition, although APAP induced autophagy at early time points in both species, as evidenced by both a decrease in abundance of SQSTM1 (at 3 h) and the conversion of LC3B-I to LC3B-II (at 3 and 6 h), the level of LC3B-I was found to be consistently higher in the rat across the timeframe of the study (Figs. 5H–J), suggestive of a greater autophagic capacity. Together, these data support a more robust activation of adaptive stress response pathways in rats compared with mice following exposure to an equivalent NAPQI insult.

#### Rats exhibit a higher basal hepatic expression of stress response pathway markers compared with mice and humans

**Figure 6.**
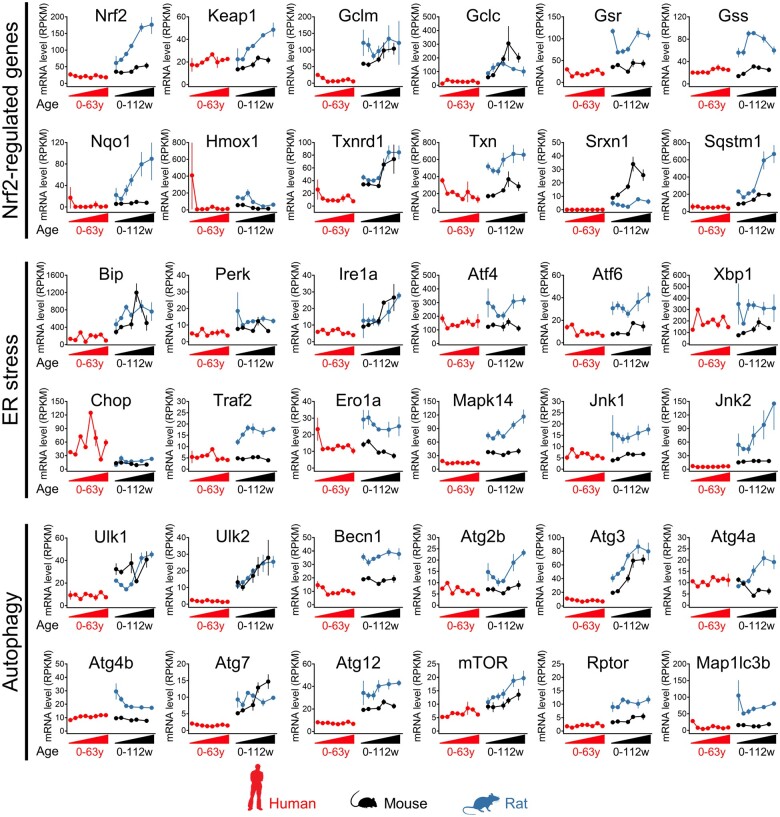
Higher basal expression levels of stress response pathway genes in the liver of rats, compared with mice and humans. Differences between species in the basal expression of Nrf2 genes and genes modulating endoplasmic reticulum (ER) stress and autophagy from birth to adulthood. mRNA levels are expressed as reads per kilo base per million (RPKM)±SD (Cardoso-Moreira *et al.*, 2019).

**Figure 7.**
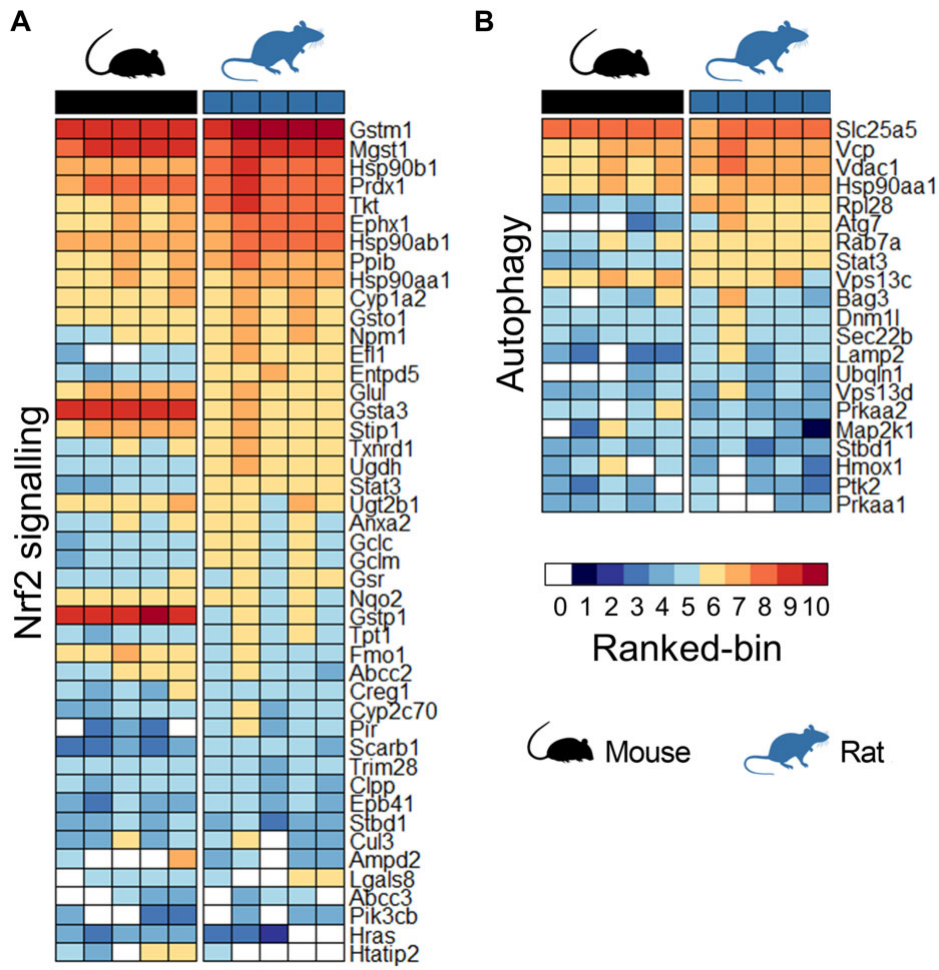
Higher basal expression levels of stress response pathway proteins in the liver of rats, compared with mice. Heatmaps showing rank-binned basal expression levels of proteins associated with the (A) Nrf2 and (B) autophagy responses in the livers of untreated animals (0 h controls, n = 5/species). Proteins with the lowest abundance were assigned to bin 1, and those with the highest to bin 10. A bin value of 0 (depicted in white) was assigned to proteins that were not detected. The difference among consecutive bins is 1.49 ± 0.16 (log_2_ expression).

**Figure 8.**
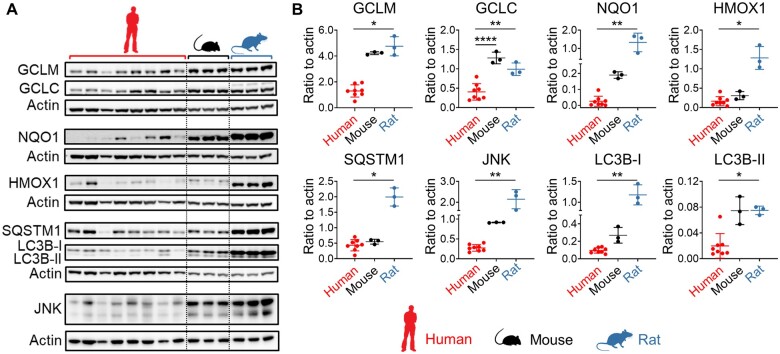
Confirmation of species differences in basal capacities of the Nrf2 and autophagy pathways. A, Protein expression levels and B, densitometric analysis of GCLM, GCLC, NQO1, HMOX1, SQSTM1, JNK, and LC3B isoforms I and II in liver tissue collected from patients undergoing liver resections (n = 8) or untreated mice (n = 3) and rats (n = 3). Protein levels were normalized to β-actin. Values are mean±SD. One-way ANOVA or Kruskal-Wallis test, as appropriate. *p* Values are denoted as *p < .05, **p < .01, and ****p < .0001, comparison as indicated.

Although our ‘omics analyses highlighted a clear difference between mice and rats in the inducible activity of several stress response pathways, the timeframe for altered expression of the associated proteins (>6 h) relative to the rapid onset of liver injury (>2 h) means that these adaptations could not have occurred swiftly enough to directly influence the different sensitivities of the species to the toxic effects of the APAP OEDs. Given the limitations of probe-based microarray and qPCR datasets for comparing absolute gene expression levels across species, we used publicly available RNA-seq (reads per kilobase million) data (Cardoso-Moreira *et al.*, 2019) to assess the basal expression levels of relevant genes in liver tissue from mice and rats, from birth to adulthood. This analysis highlighted the generally higher basal expression levels of a number of genes associated with the Nrf2 antioxidant and endoplasmic reticulum stress responses, as well as autophagic capacity, in rats compared with mice (Figure 6). These findings were supported by our proteomics data; grouping the normalized expression quantities of orthologous proteins from the 0 h control animals into ranked bins confirmed the trend of higher basal expression of Nrf2 and autophagy-associated proteins in the livers of rats, compared with mice (Figure 7, Supplementary File 4). Importantly, using the public RNA-seq data, we also found that many genes associated with these stress response pathways were expressed at a higher basal level in the liver of rats than in humans, which overall better resembled mice (Figure 6). We confirmed this at the protein level by immunoblot analysis of 0 h control tissues from animals in the APAP study, along with background liver tissue donated by 8 patients undergoing elective resection of hepatic tumors (Figs. 8A and 8B). Notably, the latter samples exhibited marked interindividual variability in the expression levels of the analyzed proteins (Figs. 8A and 8B). Although it is not possible to fully exclude differences in antibody affinity across species, the high homology (>75%) in the immunogenic sequences for the antibodies used in this study provides strong indication of comparable activity (Supplementary Table 3). Taken together, these data indicate that rats exhibit a greater basal and adaptive capacity of hepatic stress responses than mice and humans.

## Discussion

In the case of small molecule drugs, which make up a significant proportion of medicines submitted to global regulatory authorities each year, preclinical toxicity studies are expected to be performed in both a rodent and nonrodent species. Although the rat is the most commonly used rodent species, several studies have concluded that it may not be the most predictive of human drug toxicities (Greaves *et al.*, 2004; Monticello *et al.*, 2017; Olson *et al.*, 2000), highlighting the need to better understand translation of safety data across species. Differences in metabolism and disposition are known to contribute to differences in species sensitivity to drug toxicity. However, less attention has been paid to the possibility that tissue properties, such as stress response capacity, may also contribute to cases of differential species susceptibility. We have used a combination of PBPK modeling and simulation coupled to multiomics analysis to determine if differential sensitivity to APAP-induced liver injury in mice and rats is related to differences in the tissue response to equivalent burdens of the reactive intermediate NAPQI. Our study highlights the higher basal and inducible activity of the Nrf2 oxidative stress and autophagy responses in the livers of rats, compared with mice and humans. Our findings have important implications for species selection in preclinical safety testing of new medicines. Indeed, the higher stress response capacity of the rat may partly explain why it often under predicts the sensitivity of other species, including humans, to certain drug toxicities, including those associated with reactive metabolite formation and other forms of chemical stress.

Rats are well known to be relatively resistant to APAP liver toxicity, compared with mice and other sensitive species (McGill *et al.*, 2012), due in part to differences in the extent of drug bioactivation and detoxication. Indeed, Gregus *et al.* (1988) previously demonstrated that mice excrete over 50% of a dose of APAP as toxic bioactivation products (APAP-GSH and associated hydrolysis products) whereas rats excrete only 9% of APAP via these routes. As a result of this and the greater excretion of detoxication pathway metabolites (APAP-glucuronide and -sulfate) in rats, the ratio of toxic bioactivation products versus detoxication pathway metabolites was found to be 10-fold higher in mice than rats (Gregus *et al.*, 1988). Consistent with these findings, Boobis *et al.* (1986) reported that primary hepatocytes from mice and rats exhibit large differences in the extent of covalent binding and degree of toxicity following exposure to APAP *in vitro*. Notably, Tee *et al.* (1987) provided evidence that rat hepatocytes are relatively resistant to depletion of GSH and covalent protein binding upon direct exposure to NAPQI *in vitro*, compared with hepatocytes from hamsters, which are known to be highly sensitive to APAP liver toxicity. To overcome these differences in metabolic fate of the drug and enable a balanced assessment of the tissue response of mice and rats to a comparable NAPQI insult, we used PBPK modeling and simulation to support the use of 300 and 1000 mg/kg APAP as OEDs yielding an equivalent total hepatic burden of NAPQI in mice and rats, respectively. Despite this, rats remained more resistant to APAP hepatotoxicity compared with mice.

McGill *et al.* (2012) also used doses of 300 and 1000 mg/kg (albeit via intraperitoneal, rather than oral, administration) in their investigation of the biochemical events underlying the differential sensitivity of mice and rats, respectively, to APAP liver toxicity. Consistent with our findings, the authors reported a substantial increase in serum ALT activity and marked centrilobular necrosis in mice, with minimal responses observed in rats (McGill *et al.*, 2012). Total APAP-protein adducts reached similar levels in the 2 species, further confirming the equivalent degrees of chemical insult with these doses, and leading the authors to conclude that there are downstream factors responsible for the difference in sensitivity of mice and rats to APAP toxicity (McGill *et al.*, 2012). In mice, JNK has been shown to undergo early phosphorylation and translocation to mitochondria partly as a result of the initial oxidative stress provoked by APAP (Gunawan *et al.*, 2006; Saito *et al.*, 2010). Here, we have shown that p-JNK accumulates in liver samples from both mice and rats, peaking 3 h after the administration of APAP OEDs. In their study, McGill *et al.* (2012) demonstrated that APAP-protein adducts and p-JNK translocation were consistently higher in mitochondrial fractions from mouse liver, and that rats therefore do not develop the critical levels of mitochondrial dysfunction or oxidative stress associated with APAP toxicity in mice. In light of our findings, it is possible that this is due to the greater capacity of the rat liver to cope with the initial chemical insult and mitigate against the progression of downstream toxic events.

Consistent with our findings, Xu *et al.* (2021) recently reported that mice better resemble humans in terms of the basal hepatic expression of oxidative stress-related genes, whereas rats better resemble humans in terms of the expression of genes associated with lipid metabolism. Transgenic Nrf2 null mice have been reported to exhibit greater susceptibility to a large number of liver toxins, including APAP (Clarke *et al.*, 2016), whereas Kang *et al.* (2020) recently reported that the response of an Nrf2-associated gene set was typically higher in primary rat hepatocytes, compared with human, exposed to a large panel of liver toxic and safe drugs. Autophagy has been implicated in the pathogenesis of DILI (Chen *et al.*, 2014) and shown to act as a defense mechanism in APAP hepatotoxicity by removing APAP adducts (Ni *et al.*, 2016) and damaged mitochondria (Ni *et al.*, 2012). Notably, APAP protein adducts have been shown to be cleared at a faster rate in the livers of rats (Tarloff *et al.*, 1996) compared with mice (Henderson *et al.*, 2000). The Nrf2 pathway and autophagy are linked by the p62/SQSTM1 (sequestosome 1) protein, which acts as a cargo receptor for autophagic degradation of ubiquitinated targets and activates Nrf2 by interfering with its ability to bind to its inhibitor Keap1 (Copple *et al.*, 2010). SQSTM1 expression is also transcriptionally regulated by Nrf2, suggesting that SQSTM1 forms part of a regulatory feedback loop in the Nrf2 pathway (Jiang *et al.*, 2015). Hence, the differential activity of these and other cytoprotective pathways is expected to influence species sensitivity to a range of chemical insults.

In keeping with our finding of differences in the basal expression of stress response pathway components across liver tissues donated by 8 patients, Kang *et al.* (2020) recently reported quantitative differences in the drug-induced response of Nrf2-associated genes among primary hepatocytes isolated from different human donors. In addition, Liu *et al.* (2021) have provided evidence that the hepatic expression of Nrf2-regulated genes is altered in patients with certain liver diseases. Hence, although the stress response capacity of most humans likely sits within an average range, it is possible that some individuals have relatively higher or lower abilities to respond to chemical insults, which could influence interindividual sensitivity to some drug toxicities and the predictivity of relatively homogeneous animal models. Further work is required to fully understand the extent and consequences of such differences in stress response capacity between individuals.

A limitation of our *in vivo* study is that we have focused on only APAP as an exemplar hepatotoxic compound, and the C57Bl6/J and Sprague Dawley strains of mouse and rat, respectively. Harrill *et al.* (2009) have reported marked differences in sensitivity to the toxic effects of 300 mg/kg APAP across a panel of 36 inbred mouse strains, although C57Bl6/J mice were found to sit in the middle of this range, and the mechanism of APAP toxicity has been extensively investigated in this strain (McGill *et al.*, 2012). In addition, our comparison of the basal expression of stress response pathway components used public transcriptomics data from CD-1 mice, alongside Sprague Dawley rats (Cardoso-Moreira *et al.*, 2019). Furthermore, Monroe *et al.* (2020) recently demonstrated that a large number of drugs that form chemically reactive metabolites, and were terminated due to clinical liver toxicity, provoke an activation of hepatic Nrf2 signaling, despite a lack of overt liver injury, in the rat (mixture of Sprague Dawley and Han Wistar strains). Hence, although there will likely be some differences between strains, these observations indicate that, in general, the rat liver is highly resistant to chemical insult due to an increased capacity to respond to a range of stresses. Another limitation is that our study has focused on the liver, which is one of the most common targets of drug toxicity both preclinically and in patients (Wilke *et al.*, 2007). However, a broader understanding of the degree of conservation of stress responses and other key physiological traits in different organ systems across preclinical models and humans is vital to inform the selection of appropriate species for preclinical testing to best reflect the clinical setting. Such knowledge will also be useful for the parameterization of computational tools that are being designed to support risk assessment as a new drug candidate transitions from the preclinical to the clinical sphere (Weaver *et al.*, 2020), therefore improving the efficiency of the drug development process and maximizing patient safety.

## Supplementary Material

kfad085_Supplementary_Data

## Data Availability

PBPK models were deposited in BioModels (Malik-Sheriff *et al.*, 2020) and assigned the identifiers MODEL2307070001 and MODEL2307070002 for rat and mouse, respectively. The transcriptomic data discussed in this publication have been deposited in NCBI’s Gene Expression Omnibus (Edgar *et al.*, 2002) and are accessible through GEO Series accession number GSE205203 (https://www.ncbi.nlm.nih.gov/geo/query/acc.cgi?acc=GSE205203). The SWATH proteomics data have been deposited to the ProteomeXchange Consortium (http://proteomecentral.proteomexchange.org) via the PRIDE partner repository (Perez-Riverol *et al.*, 2019) with the dataset identifier PXD034438. Supplementary Files 1–4 are available at *Toxicological Sciences* online.

## Notes

The authors certify that all research involving human subjects was done under full compliance with all government policies and the Helsinki Declaration.
