# Supplementary material for: A systems approach reveals species differences in hepatic stress response capacity
Source: Toxicol Sci. 2023 Aug 30;196(1):112–25. doi: 10.1093/toxsci/kfad085 (PMC10614045; doi:10.1093/toxsci/kfad085)
Supplement: kfad085_Supplementary_Data [file kfad085_supplementary_data.zip › kfad085_Supplementary_Data/toxsci-23-0157-File010.docx]

**A systems approach reveals species differences in hepatic stress response capacity**

Giusy Russomanno, Rowena Sison-Young, Lucia A. Livoti, Hannah Coghlan, Rosalind E. Jenkins, Steven J. Kunnen, Ciarán P. Fisher, Dennis Reddyhoff, Iain Gardner, Adeeb H. Rehman, Stephen W. Fenwick, Andrew R. Jones, Guy Vermeil De Conchard, Gilles Simonin, Helene Bertheux, Richard J. Weaver, Robert L. Johnson, Michael J. Liguori, Diana Clausznitzer, James L. Stevens, Christopher E. Goldring, Ian M. Copple*.

**Table of contents**

[Supplementary materials and methods 2](#_Toc113027193)

[Supplementary tables 14](#_Toc113027194)

[Supplementary figures and figure legends 22](#_Toc113027195)

[Supplementary files 35](#_Toc113027196)

[Supplementary references 36](#_Toc113027197)

# **Supplementary materials and methods**

*PBPK modeling*

Physiologically-based pharmacokinetic (PBPK) models were constructed using the Simcyp Animal simulator (v18r2; Certara Inc.) to predict the disposition of APAP and its metabolites arising from sulfation, glucuronidation, and cytochrome P450 mediated metabolism and glutathione conjugation of the resultant reactive-metabolite, NAPQI. Details of the animal PBPK simulators and the quality assurance system used in the development of each version of the Simcyp simulator have been described previously (Jamei et al. 2009; Jamei et al. 2013; Musther et al. 2017; Rowland Yeo et al. 2010). The depletion of the phosphoadenosine 5'-phosphosulfate (PAPS) co-factor is a critical mechanism in accurately capturing the kinetics of APAP and its metabolites at higher doses. At higher doses the availability of PAPS becomes rate limiting resulting in an increased fraction of the APAP dose being metabolized through cytochrome P450 mediated metabolism producing NAPQI. A simple PAPS cofactor turnover/depletion model was implemented using the Lua (Ierusalimschy 2006) custom scripting facility implemented in Simcyp. This PAPS turnover model returns a scaling factor applied to intrinsic hepatic clearance through the sulfation pathway and capturing the shift in fraction metabolized at higher APAP concentrations.

Models were parameterized and their predictive performance verified using the observed data from the in vivo study reported here; exemplar simulations are provided in Fig. S1. The PBPK models were used to perform simulations to determine APAP OEDs (mg/kg body weight). Here the OED is defined as the orally-administered APAP dose resulting in a total (cumulative over 24 h) hepatic NAPQI burden equivalent between the species of interest. Predicted OEDs were used as doses in the in vivo time course study described below. Data from this study was then analyzed to determine the total hepatic NAPQI burden in each species. Based on glutathione depletion, total hepatic NAPQI burden was calculated by determining the loss of GSH based on the average GSH level (nmol) in control animals at 24 h (μ_GSH,vehicle_24h_) and the minimum GSH level (min_GSH,APAP_) in APAP dosed animals; this was then converted to a total hepatic burden (mg/g liver) assuming a 1:1 stoichiometry between NAPQI and glutathione (equation x). Since both treated and vehicle control animals were fasted for 16 h, GSH levels were below baseline at the start of the dosing period. We therefore assumed that GSH levels had returned to their respective baseline in both species after 24 h on resumption of feeding.

$$Total Hepatic Burden= \frac{\mu_{GSH,vehicle\_24h}-{min}_{GSH,APAP}}{{MW}_{NAPQI}\cdot{10}^{-6}}\cdot\frac{1}{LW}$$

*Equation x*

Liver weight (LW) was taken as 1.3 g and 9.0 g for mouse and rat, respectively, from Simcyp population library values (Simcyp v18r2; Certara Inc.); protein content was assumed to be 231.48 mg/g liver for both species (Wisniewski et al. 2014). See File S1 for further details of hepatic NAPQI burden calculations.

*Dose-ranging study*

Animal experiments were performed at Biologie Servier (France) in accordance with the European Council Directive 2010/63/EU on the protection of animals used for scientific purposes, the ARRIVE guidelines (Percie du Sert et al. 2020) and the Guiding Principles in the Use of Animals in Toxicology. The studies were approved by both Biologie Servier and TransQST consortium ethical review committees. Male C57Bl/6J mice (8 weeks old; 15-22 g body weight; n=5 per dose) or Sprague-Dawley rats (5 weeks old; 119-144 g body weight; n=5 per dose) were fasted for 16 h prior to dosing. APAP (Sigma-Aldrich) was solubilized in 1 % (w/v) hydroxyethylcellulose (Sigma-Aldrich). Mice were administered 150, 300, 450 or 600 mg/kg APAP by oral gavage. Rats were administered 1000, 1300 or 1600 mg/kg APAP by the same route. Vehicle control groups were administered 1 % hydroxyethylcellulose.

*Serum biomarker analysis*

Blood samples were collected from a jugular vein or abdominal aorta of isoflurane‑anesthetized animals into tubes with a serum separator. Blood samples were centrifuged at top speed after clotting and analyzed using a Cobas c501 analyzer (Roche). Alanine aminotransferase (ALT) and aspartate aminotransferase (AST) activities were measured kinetically at 37 °C according to the International Federation of Clinical Chemistry recommendations without Pyridoxal Phosphate (Roche). Total bilirubin concentration (TBIL) was measured using 3,5-dichlorophenyl diazonium (Roche).

*Histopathological assessment*

For each animal, the entire liver (gall bladder was carefully removed for the mouse) was rapidly excised and briefly rinsed in DNase/RNase -free distilled water. The left liver lobe was then separated and preserved in 10 % formalin. Fixed samples were mounted in paraffin wax, cut at approximately 4 µm in thickness, stained with hematin-eosin saffron (HES) and evaluated by two independent veterinary pathologists. The extent of centrilobular hepatocellular necrosis/degeneration was assigned a consensus semi-quantitative score as follows: 0, no degeneration/necrosis observed; 1, minimal degeneration/necrosis of individual or small groups of hepatocytes; 2, mild degeneration/necrosis that bridges some centrilobular zones; 3, moderate degeneration/necrosis that completely bridges centrilobular zones; 4, marked degeneration/necrosis that bridges centrilobular zones and extends into midzone areas; 5, severe degeneration/necrosis involving all hepatic lobular zones.

*Liver glutathione measurement*

Liver tissue was homogenized in buffer (pH 7.4) comprising 143 mM NaH_2_PO_4_, 8 mM EDTA and 1.3 % sulfosalicylic acid, using an oscillating mill at 30 oscillations/sec for 3 min. The homogenates were then centrifuged (18,000 g for 5 min at 4 **°**C) and the supernatants collected. Total glutathione (GSH) was measured as described previously (Vandeputte et al. 1994). The reaction was followed kinetically at 415 nm. Sample readings were interpreted against a GSH calibration curve and normalised to protein content, which was determined from the sample pellets (dissolved in 1 M NaOH at 60 **°**C) generated during the homogenization step, using the bicinchoninic acid method (Sigma-Aldrich).

*Immunoblotting*

Snap frozen liver tissue (30-40 mg) was homogenized in radioimmunoprecipitation buffer (Sigma Aldrich). Liver homogenate (20 μg) was separated by SDS-PAGE under reducing conditions, transferred to nitrocellulose membranes and subjected to immunoblot analysis as previously described (Copple et al. 2010). Details of all antibodies used in this study are provided in Table S2. Immunoreactive bands were visualized using a ChemiDoc Imaging System (Bio-Rad) and volumes were quantified using Image Lab Version 6.1.0 (Bio-Rad), with normalization to β-ACTIN. Total protein normalization was also used to exclude the effect of APAP treatment on β-ACTIN expression, and yielded similar results (data not shown). For the representative blots, a pool of all samples for each treatment group and time point were loaded onto the gel. To allow for a fair comparison across species when mouse and rat samples were run on separate gels due to the number of time points/treatment conditions within an experiment, representative samples from the other species were added to each gel as internal controls, and the chemiluminescence signals associated with the two gels were quantified together.

To evaluate the affinity of each antibody for different species’ proteins, we identified the immunogen sequence (when provided by the supplier) and used NCBI and UniProt BLAST (Johnson et al. 2008; Zaru et al. 2023) to calculate the percentage of homology across species (Table S3). For all the primary antibodies used in this study the immunogen sequences showed high homology across species (>75%), providing strong indication of similar affinity in different organisms.

*Pharmacokinetic analysis*

Plasma samples (collected in heparin-coated tubes and stored at -80 °C until analysis) were thawed at room temperature and centrifuged (3000 g for 5 min at 4 °C). Each sample (10 μL) was then spiked with 10 μL internal standard (8 µg/mL for APAP d4 and APAP Gluc d3, 9 µg/mL for APAP Sul d3 and 0.6 µg/mL for APAP NAC d5, APAP Cys d5 and APAP GSH d3; all in MeOH) or MeOH alone as blank. Proteins were precipitated from each sample by adding 180 μL MeOH, mixed (1500 rpm for 5 min), stored at -20 °C for 20 min, centrifuged (3000 g for 5 min at 4 °C) and the supernatants collected. Each sample was further diluted with 600 μL water, mixed (800 rpm for 2 min), centrifuged (3000 g for 5 min at 4 °C) and the supernatant used for injection into the UPLC-MS/MS system. The samples were analyzed using an UPLC-I Class system (Waters) coupled to an API 4000 QTrap mass spectrometer (Sciex). Separation was performed using an Acquity UPLC HSS T3, 1.8 μm, 100 x 2.1 mm with a 0.2 μm pre-filter (Phenomenex) and a column oven set to 40 °C. The injection volume was 2 μL and flow rate 0.6 mL/min. The mobile phases and gradient program are summarized in Table S4. Mass spectrometric analysis was performed in either positive or negative ion mode, as detailed in Tables S5 and S6.

*Transcriptomic analysis*

Total RNA was isolated from liver tissue collected in RNAlater^®^ stabilizing reagent immediately prior to dosing (0 h) or at 3, 6, 9, and 24 h after administration of APAP or vehicle. Frozen liver samples were homogenized in Qiazol Lysis Reagent (Qiagen). Total RNA was purified following the manufacturer’s instructions. The RNA integrity in representative samples was verified using an Agilent BioAnalyzer. Total RNA (5 µg) was processed per the standard Affymetrix protocol for microarray target preparation. Resulting cRNA was fragmented and hybridized onto Affymetrix GeneChips, which were then washed, stained, and scanned using the standard Affymetrix procedure (Affymetrix GeneChip^®^ Mouse Genome 430 2.0 Array or Affymetrix GeneChip^®^ Rat Genome 230 2.0 Array). Quality control and low level analysis of raw fluorescence intensities was performed using the affy package (v1.64.0) in R v3.6.1 (Gautier et al. 2004). Mouse data were processed using RMA normalization and BrainArray CDF Version 24 (Dai et al. 2005). Rat data were processed using RMA normalization and BrainArray CDF Version 19, which gives 100 % coverage on the DILI TXG-MAPr tool (https://txg-mapr.eu/) used for Weighted Gene Co-Expression Network Analysis (WGCNA). Differential expression analyses were carried out using the limma package (v3.42.2) in R (Ritchie et al. 2015). Orthologous genes between rat and mouse represented in the Rat Genome Database (RGD) were considered for comparison of the transcriptomic changes. Genes were considered differentially expressed when the adjusted P value (Benjamini-Hochberg correction) was less than 0.05 and there was at least a 1.5-fold change in expression. The full list of significant genes at each time point was analyzed for membership in co-expressed gene sets using WGCNA, and an eigengene score (EGs, or module score) which summarizes the log_2_ fold change of the respective constituent genes, was calculated for each module as previously described (Sutherland et al. 2018). The average absolute EGs was calculated by averaging the absolute scores across all the modules in each species at each time point. The list of the top 50 most significant genes in each module (ordered by significance) and their relative log_2_ fold-change can be found in File S3.

*Pathway analysis*

ClusterProfiler package (v3.18.0) (Yu et al. 2012) was used for gene set enrichment analysis (GSEA) on the lists of differentially expressed genes (DEGs) ranked by log_2_ fold-change. The number of permutations was set to 10,000. To remove differences in the setSize, genome wide annotation for Rat (package ‘org.Rn.eg.db’) was used for both species. Gene ontology (GO) biological processes (Ashburner et al. 2000) were considered significantly enriched with a FDR-corrected P_adj_< 0.05 and absolute Normalised Enrichment Score (NES) >1.5. The full list of significantly enriched GO biological processes in both species at all time points is provided in File S2. Genes that were differentially expressed in either species at any time point within the lists of enriched genes in selected GO biological processes were used to generate heatmap plots showing changes at a gene level between species (data expressed as log_2_ fold-changes *versus* time-matched vehicle controls animals).

*qPCR analysis*

RNA (500 ng) was reverse transcribed using a LunaScript RT SuperMix kit (New England Biolabs) according to the manufacturer’s instructions in a total 20 µL reaction mix. qPCR was performed using Luna Universal qPCR Master Mix (New England Biolabs) on an ABI ViiA-7 Thermocycler (Applied Biosystems). Primer sequences for mouse and rat genes are detailed in Table S7. For relative quantification, data were analyzed using the 2^-ΔΔCt^ method. The selection of *Gapdh* as the endogenous normalization control was based on analysis of the transcriptomic data using NormFinder (Andersen et al. 2004), which ranked homologous genes with a coefficient of variation < 1% across treatments and time points, based on the calculated stability values.

*Proteomics sample preparation*

Liver tissue (100-200 mg) was lysed by sonication on ice in 300 µL of 7 M urea, 2 M thiourea, 40 mM tris (pH 7.5) and 4 % w/v CHAPS buffer. After centrifugation (14,000 rpm for 10 min at 4 °C), the supernatant was collected and protein concentration was determined by Bradford assay. Next, 200 µg protein was reduced with 5 mM dithiothreitol at 37 °C for 30 min, alkylated with 0.25 M iodoacetamide, and incubated at 37 °C for 3 h with Trypsin/Lys-C (Promega) at a 25:1 protein:protease ratio (w/w). The reaction mix was then diluted 1:10 with 50 mM ammonium bicarbonate in order to reduce the urea concentration to less than 1 M, and incubated overnight at 37 °C. Peptides were then diluted to 5 mL with 10 mM potassium dihydrogen phosphate/25 % acetonitrile (ACN) and acidified to pH ≤ 3 with phosphoric acid prior to cation exchange chromatography. In order to generate the reference spectral libraries, a pool of 20 samples/species representative of all the experimental conditions was prepared. An aliquot of 1.5 mg of the pool was pre-fractionated on a Polysulfoethyl A column (200 × 4.6 mm, 5 μm, 300 A; Poly LC) at a flow rate of 1 mL/min. For individual samples, detergents and undigested protein were removed using a Bio-Scale Mini Macro-Prep High S 1 mL cartridge (Bio-Rad) on an Agilent 1100 HPLC system at 1 mL/min. The mobile phases consisted of 10 mM KH_2_PO_4_/25 % ACN w/v, pH 3 (phase A), and 10 mM KH_2_PO_4_/1 M KCl/25 % ACN w/v, pH 3 (phase B). A gradient from 0-50 % B in 90 min was applied to the Polysulfoethyl A column. Peptides were eluted from the cartridge using a gradient time program set as follows (phase B): 0 %; 0-0.5 min, 0-15 %; 0.5-5 min, 15 %; 5-5.10 min, 15-50 %; 5.10-7 min, 50 %; 7-7.10 min, 50-0 %; 7.10-10 min, 0 %. The system was then washed with 100 % phase A for 5 min after each sample to prevent sample carryover. Fractions of 1 mL were dried in a SpeedVac Concentrator Plus (Eppendorf), then reconstituted in 1 mL of 0.1 % trifluoroacetic acid and desalted using an Agilent Macroporous Reversed-Phase C18 column (4.6 × 50 mm mRP-C18, Agilent) on a 1260 Infinity HPLC system (Agilent).

*Data-dependent acquisition (DDA) and data analysis*

Desalted fractions were reconstituted in 0.1 % formic acid and 0.5-1 μg of sample was loaded onto a UPLC Symmetry C18 nanoAcquity Trap Column (Waters). After a 10 min wash with 2 % ACN/0.1 % formic acid, the trap was switched in-line with a peptide BEH C18 nanoAcquity column (1.7 µm, 75 µm x 250 mm; Waters) and a gradient of 2-50 % ACN/0.1 % formic acid was applied over 120 min at a flow rate of 300 nL/min. DDA was performed on a Triple TOF 6600 (Sciex) in positive ion mode with a target of 25 MS/MS per cycle (2.8 sec cycle time), exclusion of former target ions for 20 sec, and using a mass range of 400-1800 for MS and 100-1500 for MS/MS. The data were searched using ProteinPilot 5.0 (Sciex) and the Paragon algorithm (Sciex) against the UniProtKB database (Mus musculus UP000000589, gene count 22,001, 55,366 proteins, last modified March 2021; Rattus norvegicus UP000002494, gene count 21,588, 29,934 proteins, last modified March 2021). Proteotypic peptides with no modifications except carbamidomethylation of cysteine residues were included in the library, so a ‘rapid’ search of the data was performed using ProteinPilot. Mass tolerance for precursor and fragment ions was 10 ppm. An FDR of 1 % was applied using the reversed database as decoy. This resulted in 5,253 and 5,823 proteins being included in the mouse and rat libraries, respectively.

*SWATH acquisition and data analysis*

The same sample loading and chromatography conditions as described above were used for the individual sample acquisitions. Sequential Window Acquisition of all Theoretical Mass Spectra (SWATH) acquisitions were performed using 100 SWATH windows of variable effective isolation width to cover a mass range of 400-1600 m/z. The total cycle time was 3.1 sec. To account for batch effects, a pool of 20 samples representative of all the experimental conditions and a yeast digest quality control (Promega) were included with the samples in each batch. SWATH data were aligned with the spectral libraries using DIA-NN (v1.8) (Demichev et al. 2020) with default settings in ‘robust LC (high accuracy)’ mode and annotated using the reference proteomes (UP000000589 and UP000002494) downloaded as FASTA files. Mass tolerances were determined automatically in DIA-NN for each run separately (Unrelated runs option). Match-between-runs was enabled to re-process the same dataset using a spectral library generated from the data-independent acquisition (DIA) data. Only proteins identified with proteotypic peptides and protein q-value below 0.01 were considered. Normalization and differential expression analyses were carried out using the DEqMS package (v1.8.0) in R (Zhu et al. 2020). Protein quantities were log_2_ transformed and normalised using the equalMedianNormalization function, and Limma batch effect correction was applied. Ingenuity Pathway Analysis software (IPA; Qiagen) was used to investigate enriched canonical pathways and toxicity functions (IPA-Tox) in the two species. Comparison analyses were performed to evaluate changes in the z-score for each function over time and across species and reveal biological pathways underlying toxicity-specific phenotypes. Since the mouse and rat SWATH datasets were analyzed separately, log_2_ transformed normalised protein expression values of orthologous proteins from the 0 h control animals (untreated) were ranked and grouped into 10 bins within each dataset for comparison of basal protein abundances across species (Wang et al. 2022). Proteins with the lowest protein abundance values were assigned to bin 1, whereas those with the highest abundance values were assigned to bin 10. Proteins that were not detected (NA, not available) were assigned a bin value of 0. See File S4 for more details.

*Feed comparison study*

All experiments were performed in accordance with a license granted under the UK Animals (Scientific Procedures) Act 1986 and were approved by the University of Liverpool Animal Ethics Committee. All animals received humane care according to the criteria outlined in the Guide for the Care and Use of Laboratory Animals. Male C57Bl/6J mice (7-8 weeks old) and male Sprague-Dawley rats (7-8 weeks old) were supplied by Charles River (UK). After the acclimatization period (1 week), animals (n=4/group) were assigned to either RM1 (RM1P-E-FG, Special Diet Services, UK) or A04 (DS-SAFE-A04, Safe, France) diet for 7 days. All animals received food and water *ad libitum*. At the end of the study, the animals were culled via exposure to a rising concentration of CO_2_, and liver tissue was excised and snap-frozen for later processing. RNA was extracted using a Monarch total RNA miniprep kit (New England Biolabs, UK) following manufacturer’ instructions. Input RNA was reverse transcribed and qPCR was performed as described in the main manuscript.

# Supplementary tables

**Table S1.** Details of patients donating liver tissue as part of planned liver resection.

| **Donor ID** | **Sex** | **Age** | **BMI** | **Indication** | **Underlying liver disease** |
| --- | --- | --- | --- | --- | --- |
| S217 | 78 | F | 30.3 | CCA | Mild macrovesicular steatosis |
| S200 | 70 | M | 29.9 | HCC | None |
| S005 | 60 | F | 28.7 | CCA | Moderate microvesicular steatosis |
| S006 | 48 | M | 24.0 | CCA | Non-cirrhotic fibrosis |
| S285 | 71 | M | 21.8 | CCA | None |
| S205 | 73 | F | 28.9 | CRLM | Mild macrovesicular steatosis |
| S002 | 56 | F | 21.1 | CCA | None |
| S201 | 69 | M | 32.4 | HCC | Non-cirrhotic fibrosis/macrovesicular steatosis |

BMI, body mass index; HCC, hepatocellular carcinoma; CCA, cholangiocarcinoma; CRLM, colorectal cancer liver metastases.

**Table S2.** List of antibodies used for western blot experiments.

| **Antigen** | **Host** | **Dilution factor** | **Company** | **Cat. n.** |
| --- | --- | --- | --- | --- |
| **GCLC** (Glutamate-Cysteine Ligase Catalytic Subunit) | Rabbit | 1:5000 | Abcam (UK) | ab41463 |
| **GCLM** (Glutamate-Cysteine Ligase Modifier Subunit) | Rabbit | 1:5000 | Abcam (UK) | ab126704 |
| **HMOX1** (Heme Oxygenase 1) | Rabbit | 1:5000 | Abcam (UK) | ab13243 |
| **LC3B** (Autophagy marker Light Chain 3 isoform B) | Rabbit | 1:750 | Cell Signaling Technology (US) | #2775 |
| **NQO1** (NAD(P)H dehydrogenase quinone 1) | Goat | 1:2000 | Abcam (UK) | ab2346 |
| **p-SAPK/JNK** (Phospho-stress-activated protein kinases/Jun amino-terminal kinases) | Rabbit | 1:1000 | Cell Signaling Technology (US) | #4668 |
| **SAPK/JNK** (Stress-activated protein kinases/Jun amino-terminal kinases) | Rabbit | 1:1000 | Cell Signaling Technology (US) | #9252 |
| **SQSTM1** (Sequestosome 1) | Rabbit | 1:2500 | Sigma-Aldrich (UK) | P0067 |
| β-ACTIN | Mouse | 1:10000 | Abcam (UK) | ab6276 |
| Rabbit IgG | Goat | 1:5000 | Sigma-Aldrich (UK) | A9169 |
| Mouse IgG | Rabbit | 1:5000 | Sigma-Aldrich (UK) | A9044 |
| Goat IgG | Rabbit | 1:5000 | Agilent/Dako (US) | P044901-2 |

**Table S3.** Immunogen homology across different species for the primary antibodies used in western blot experiments.

|  |  |  | **% Immunogen homology**  **(UniProt ID)** | | |
| --- | --- | --- | --- | --- | --- |
| **Antigen** | **Tested Reactivity (Supplier)** | **Immunogen** | **H** | **M** | **R** |
| **GCLC** | H, M, R | Synthetic peptide corresponding to Human GCLC aa 50-150 (UniProt P48506). Inferred reference sequence:  EVEYMLVSFDHENKKVRLVLSGEKVLETLQEKGERTNPNHPTLWRPEYGSYMIEGTPGQPYGGTMSEFNTVEANMRKRRKEATSILEENQALCTITSFPRL | 100  (P48506) | 91.1  (P97494) | 90.1  (P19468) |
| **GCLM** | H, M, R | Synthetic peptide within Human GCLM aa 50-150 (UniProt P48507). Inferred reference sequence:  TLNEWSSQINPDLVREFPDVLECTVSHAVEKINPDEREEMKVSAKLFIVESNSSSSTRSAVDMACSVLGVAQLDSVIIASPPIEDGVNLSLEHLQPYWEEL | 100  (P48507) | 96  (O09172) | 95  (P48508) |
| **HMOX1** | H, M, R | Recombinant Rat HMOX1 aa 1-266 (UniProt P06762), lacking the membrane spanning region (aa 267-289). Inferred reference sequence:  MERPQLDSMSQDLSEALKEATKEVHIRAENSEFMRNFQKGQVSREGFKLVMASLYHIYTALEEEIERNKQNPVYAPLYFPEELHRRAALEQDMAFWYGPHWQEAIPYTPATQHYVKRLHEVGGTHPELLVAHAYTRYLGDLSGGQVLKKIAQKAMALPSSGEGLAFFTFPSIDNPTKFKQLYRARMNTLEMTPEVKHRVTEEAKTAFLLNIELFEELQALLTEEHKDQSPSQTEFLRQRPASLVQDTTSAETPRGKSQISTSSSQT | 79.5  (P09601) | 93.2  (P14901) | 100  (P06762) |
| **LC3B** | H, M, R | Not provided. Inferred reference sequence for Human LC3B aa 1-125 (UniProt Q9GZQ8):  MPSEKTFKQRRTFEQRVEDVRLIREQHPTKIPVIIERYKGEKQLPVLDKTKFLVPDHVNMSELIKIIRRRLQLNANQAFFLLVNGHSMVSVSTPISEVYESEKDEDGFLYMVYASQETFGMKLSV | 100  (Q9GZQ8) | 95.2  (Q9CQV6) | 96  (Q62625) |
| **NQO1** | H, R | Synthetic peptide:  C-SIPTDNQIKARK | 100  (P15559) | 91.7  (Q64669) | 91.7  (P05982) |
| **SAPK/JNK** | H, M, R | Not provided. As the antibody detects endogenous levels of total JNK1, JNK2 or JNK3 protein, the sequence of Human JNK1 aa 1-427 (UniProt P45983-1) was used as a comparator for all other isoforms/family members:  MSRSKRDNNFYSVEIGDSTFTVLKRYQNLKPIGSGAQGIVCAAYDAILERNVAIKKLSRPFQNQTHAKRAYRELVLMKCVNHKNIIGLLNVFTPQKSLEEFQDVYIVMELMDANLCQVIQMELDHERMSYLLYQMLCGIKHLHSAGIIHRDLKPSNIVVKSDCTLKILDFGLARTAGTSFMMTPYVVTRYYRAPEVILGMGYKENVDLWSVGCIMGEMVCHKILFPGRDYIDQWNKVIEQLGTPCPEFMKKLQPTVRTYVENRPKYAGYSFEKLFPDVLFPADSEHNKLKASQARDLLSKMLVIDASKRISVDEALQHPYINVWYDPSEAEAPPPKIPDKQLDEREHTIEEWKELIYKEVMDLEERTKNGVIRGQPSPLGAAVINGSQHPSSSSSVNDVSSMSTDPTLASDTDSSLEAAAGPLGCCR | 77.6-100* | 80.8-100* | 79.5-98.8* |
| **SQSTM1** | H, M, R | Synthetic peptide corresponding to Human SQSTM1 aa 256-269 (UniProt Q13501). Inferred reference sequence:  IDVEHGGKRSRLT | 100  (Q13501) | 100  (Q64337) | 100  (O08623) |
| **β-ACTIN** | H, M, R | Synthetic peptide:  DDDIAALVIDNGSGK | 92.9  (P60709) | 92.9  (P60710) | 92.9  (P60711) |

aa, amino acid; H, human; M, mouse; R, rat.

*Depending on family member and isoform (range, min-max).

**Table S4.** LC-MS/MS conditions for pharmacokinetic analysis of APAP metabolites in plasma. Details of the liquid chromatography conditions used for analyte separation.

| Time (min) | 0 | 0.5 | 1.85 | 1.90 | 2.50 | 4.0 | 5.0 | 5.1 | 6 | 6.1 | 8.1 |
| --- | --- | --- | --- | --- | --- | --- | --- | --- | --- | --- | --- |
| % A (H_2_O + 0.1 % HCOOH) | 95 | 95 | 93 | 92 | 90 | 84 | 75 | 5 | 5 | 95 | 95 |
| % B (Methanol + 0.1 % HCOOH) | 5 | 5 | 7 | 8 | 10 | 16 | 25 | 95 | 95 | 5 | 5 |
| Curve | - | 6 | 6 | 6 | 6 | 6 | 6 | 6 | 6 | 6 | 6 |

**Table S5.** Mass spectrometric parameters used in either negative or positive mode.

| Ionization mode: | Positive in period 2 and negative in periods 1 and 3 |
| --- | --- |
| Probe: | Horizontal: 5mm, Vertical: 2mm, Protrusion: 0.5mm |
| Curtain gas (CUR): | Nitrogen, 35psi |
| Collision gas (CAD): | Nitrogen, Medium (negative) and 6 (positive) |
| Ion Spray voltage (IS): | -4500 V (negative) and 5500 V (positive) |
| Temperature (TEM): | 500 °C |
| Ion source gas 1(GS1): | Air, 50 psi |
| Ion source gas 2 (GS2): | Air, 50psi |
| Entrance potential (EP): | 10 V |
| Interface heater (ihe): | On |
| Period 1: | 2.574 min |
| Period 2: | 1.205 min |
| Period 2: | 4.250 min |
| Total Run: | 8.030 min |

**Table S6.** MRM parameters for the absolute quantification of each APAP metabolite.

| **Compounds** | **MRM Transition** | **Mode** | **DP (V)** | **CE (eV)** | **CXP (V)** | **Dwell (msec)** | **Retention times** | **Period** |
| --- | --- | --- | --- | --- | --- | --- | --- | --- |
| APAP Gluc | 326.1 >149.9 | Negative | -75 | -38 | -11 | 50 | 1.64 min | 1 |
| APAP Gluc d3 | 329.1 > 153.0 | Negative | -75 | -40 | -7 | 50 | 1.62 min | 1 |
| APAP Sul | 230.0 >149.8 | Negative | -70 | -28 | -9 | 50 | 1.98 min | 1 |
| APAP Sul d3 | 232.9 > 152.8 | Negative | -60 | -28 | -11 | 50 | 1.96 min | 1 |
| APAP Cyst | 269.0 > 181.9 | Negative | -65 | -26 | -13 | 50 | 2.30 min | 1 |
| APAP Cyst d5 | 274.0 > 186.9 | Negative | -60 | -22 | -9 | 50 | 2.27 min | 1 |
| APAP | 152.1 > 110.1 | Positive | 61 | 23 | 18 | 100 | 2.71 min | 2 |
| APAP d4 | 156.1 > 114.1 | Positive | 61 | 23 | 18 | 100 | 2.68 min | 2 |
| APAP NAC | 311.0 > 181.9 | Negative | -60 | -24 | -9 | 100 | 4.84 min | 3 |
| APAP NAC d5 | 315.9 > 186.9 | Negative | -60 | -24 | -9 | 10 | 4.82 min | 3 |
| *APAP GSH | 457.1 > 328.1 | Positive | 71 | 23 | 10 | 50 | 3.57 min | N/A |
| *APAP GSH d3 | 460.1 > 331.1 | Positive | 71 | 23 | 10 | 50 | 3.5 min | N/A |

*Metabolites analyzed separately from the main run under positive mode during the entire LC-MS/MS run.

**Table S7.** Details of specific mouse and rat primers used in real-time qPCR experiments.

| **Gene** | **Primer** | **Mouse sequence (5’→3’)** | **Region amplified** | **Rat sequence (5’→3’)** | **Region amplified** |
| --- | --- | --- | --- | --- | --- |
| **Abcb1a** | Fwd | CGGAGTCAGACAGAACAAGAAGA | NM_011076.3 | CAGCATCGCCGAGAACATTG | NM_133401.1 |
|  | Rev | AATGCTTCCAGGCATAAGCG |  | CGACAGCTGAGTCCCTTTGT |  |
| **Abcc3** | Fwd | AAGACTCGGGTGCTGGTAAC | NM_001363187.1 | TCCCACCTGAGTGCATTTGT | NM_080581.1 |
|  | Rev | TGCAAGGCTGCTTCATGGTC |  | TCAAACTGGGTACGGATGGT |  |
| **Atg101** | Fwd | CTCGGTGCGGGTCCAAATAG | NM_026566.2 | AGTGGGTCTTGACCGAACTG | NM_001007659.2 |
|  | Rev | CCCTTCTTCCCTAACGCGAA |  | CGCAAACCAAGTGAGGTGAG |  |
| **Atg12** | Fwd | TAAACTGGTGGCCTCGGAAC | NM_026217.3 | TGGGGATGAGCCACAAATGAA | NM_001038495.1 |
|  | Rev | ATCCCCATGCCTGGGATTTG |  | AGTCTCTTCCCACAGCATCAA |  |
| **Atg14** | Fwd | ACTGCTGGGCTCGTGTTTTA | NM_172599.4 | CCAGAGCGGTGATTTCGTCT | NM_001107258.1 |
|  | Rev | TTGCTGTAGGCGGTAGTTGG |  | CGTTTTCCTTCCATGGCCTTT |  |
| **Atg16l2** | Fwd | AGAAGCTATGCACTGGCAGG | NM_001111111.1 | CAAAAGGCGCTCTTCTTGGAG | NM_001191560.1 |
|  | Rev | CATTAACAGCAGTGCAGTGGG |  | TGGCTAGCAGTTCAGCCTTC |  |
| **Bbc3** | Fwd | ATAGAGCCACATGCGAGCG | NM_133234.2 | AACTAGGTGCCTACACCCGT | NM_173837.2 |
|  | Rev | GTGGGTTGCTATTGAGGCAC |  | TGGTGCAGAAAAAGTCCCCC |  |
| **Edem1** | Fwd | GCGCTTCAAAATAATGCCCG | NM_138677.2 | GGGTGTGTGTGAGGACGATT | NM_001305279.1 |
|  | Rev | CCGAAGACCAACCAGAGCAC |  | GCAAACTTCCCATTCGCAGG |  |
| **Gapdh** | Fwd | TGTCCGTCGTGGATCTGAC | NM_001289726.1 | TTCAACGGCACAGTCAAGGC | NM_017008.4 |
|  | Rev | CCTGCTTCACCACCTTCTTG |  | TCACCCCATTTGATGTTAGCG |  |
| **Gclc** | Fwd | ATGATAGAACACGGGAGGAGAG | NM_010295.2 | AAAGCTTGGCTTAATCTACAGTTCA | NM_012815.2 |
|  | Rev | TGATCCTAAAGCGATTGTTCTTC |  | GGGAATAGTCTGCATCCTGCTT |  |
| **Gclm** | Fwd | AATCAGCCCCGATTTAGTCAG | NM_008129.4 | TCAAGCTCACAACTCAGGGG | NM_017305.2 |
|  | Rev | CGATCCTACAATGAACAGTTTTGC |  | CGCCTCAGTGACGCTTTTTG |  |
| **Hmox1** | Fwd | GTCAAGCACAGGGTGACAGA | NM_010442.2 | GGAAAGCAGTCATGGTCAGTCA | NM_012580.2 |
|  | Rev | ATCACCTGCAGCTCCTCAAA |  | CCCTTCCTGTGTCTTCCTTTGT |  |
| **Hsf1** | Fwd | CAACTGCCTTCATTGACTCCA | NM_001331152.1 | CCTCCTGTGTGTTTCCTCCTG | NM_024393.1 |
|  | Rev | GGCTCCGGTTGTGTCCATAG |  | GCCAGATTGGTCCCCTAAAG |  |
| **Il1r1** | Fwd | TGCCTCCCAGTAAAACAGTC | NM_001123382.1 | CCTCTGCCTCTTGACGATGG | NM_013123.3 |
|  | Rev | TCTCTTCCCAATCCAGTTCC |  | TGGTATGTGTAGGACGTGCG |  |
| **Keap1** | Fwd | CACAGCAGCGTGGAGAGA | NM_016679.4 | ATGTGATGAACGGGGCAGTC | NM_057152.2 |
|  | Rev | CAACATTGGCGCGACTAGA |  | AAGAACTCCTCCTCCCCGAA |  |
| **Lgals3** | Fwd | GAGTACTAGAAGCGGCCGAG | NM_001145953.1 | CAGTGCCCTACGATATGCCC | NM_031832.1 |
|  | Rev | CCTGATTAGTGCTCCACCCG |  | TGGGCTTCACTGTGCCTATG |  |
| **Mafg** | Fwd | ATGACGACCCCCAATAAAGGA | NM_010756.3 | GCCTTAAAGGTGAAGCGGGA | NM_022386.2 |
|  | Rev | CACCGACATGGTTACCAGC |  | AGGTGCTGGTTCAACTCTCG |  |
| **Nle1** | Fwd | CGTTCTACGTCCACGATGCT | NM_145431.2 | TGCTGAGATTGTGTCCTCGC | NM_001127534.1 |
|  | Rev | CAGGTACTTTCCTGTGGGGC |  | GAAATGACGGCTTCGCTGTG |  |
| **Nqo1** | Fwd | TTTAGGGTCGTCTTGGCAAC | NM_008706.5 | GTTTGCCTGGCTTGCTTTCA | NM_017000.3 |
|  | Rev | GTCTTCTCTGAATGGGCCAG |  | ACAGCCGTGGCAGAACTATC |  |
| **Nrf2** | Fwd | CATGATGGACTTGGAGTTGC | NM_010902.5 | CTTGCTCTTGGGAACAAGGAA | NM_001399173.1 |
|  | Rev | CCTCCAAAGGATGTCAATCAA |  | CGACAGAAACCTCCATCTTCTG |  |
| **Prkaa1** | Fwd | TCTTCTCCTTAACTCCTCCCTC | NM_001013367.3 | TTCGGGAAAGTGAAGGTGGG | NM_019142.2 |
|  | Rev | TGTGTGTGGCATTCCATTCATC |  | TCTCTCTGCGGATTTTCCCG |  |
| **Sesn2** | Fwd | ACTGCGTCTTTGGCATCAGA | NM_144907.1 | TCCCCCTAAGCCTGTTCTGT | NM_001109358.2 |
|  | Rev | GTCTTCTCAGGGTAGCAGGC |  | CCACCAGGCATGGGAGAAAA |  |
| **Slc25a37** | Fwd | GGGCCTGAACGTGATGATGA | NM_026331.3 | CCCCTTCCAATCTATCCACTTC | NM_001013996.1 |
|  | Rev | ACTCCCAGCTACCCCATTAG |  | TCCTGAGATGATGTGAGACTG |  |
| **Smad7** | Fwd | TTTCCTCCTCCTTTCTCGTC | NM_001042660.1 | TTTTTCCCCCCACCCTTCCAAC | NM_030858.1 |
|  | Rev | CAAAACACACACACACAACC |  | AACACACCACCTTCTCGCAC |  |
| **Socs3** | Fwd | CACAGCCTTTCAGTGCAGAGTA | NM_007707.3 | CCCCGCTTTGACTGTGTACT | NM_053565.1 |
|  | Rev | CGTAAGAGCAGGCGAGTGT |  | AAAGGAAGGTTCCGTCGGTG |  |
| **Srxn1** | Fwd | ACTATTCCTTTGGGGGCTGC | NM_029688.5 | ACCTCCTGATACCCCACTCC | NM_001047858.3 |
|  | Rev | GCTTGGCAGGAATGGTCTCT |  | GGAACCCCCTCATTCTTGGG |  |
| **Tgfbr2** | Fwd | CACGTTCCCAAGTCGGATGT | NM_009371.3 | CCCCCGTTTGGTTCCAGAGT | NM_031132.3 |
|  | Rev | GTTTCAGTGGATGGATGGTCCT |  | GGTCTCTCAGCACGTTGTCT |  |
| **Trib3** | Fwd | CCTGCGTGATGACTGGATCA | NM_175093.2 | GGGACTCCGAGATAGGCTCA | NM_144755.2 |
|  | Rev | CCGCTTTGCCAGAGTAGGAT |  | AGATGTGGCTCGCATCTTGT |  |
| **Txnrd1** | Fwd | TGTCAGGACAGCCAGTACTCTG | NM_001042513.1 | ACATGGAATTGGAATTTGGGTT | NM_031614.3 |
|  | Rev | CGGTCATGTAACCTAGGAGCTAA |  | CAAAGGAGTGGACTGTTGAGTTT |  |

# Supplementary figures and figure legends


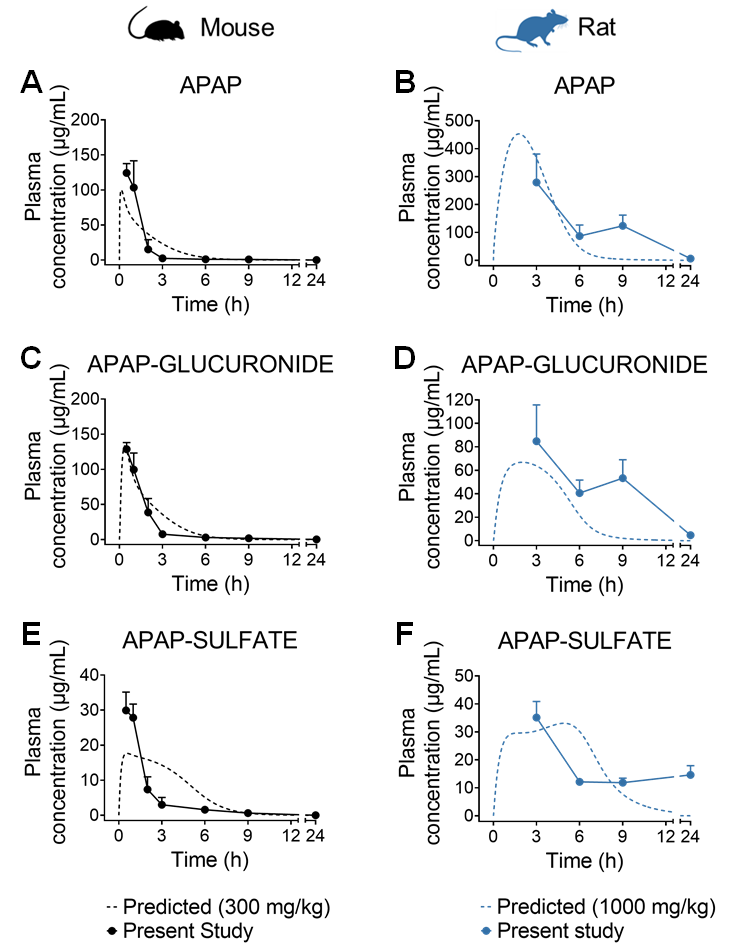


**Figure S1.** Performance verification of physiologically-based pharmacokinetic (PBPK) models for simulation of APAP (**A-B**), glucuronidated metabolites (**C-D**), and sulfated metabolites (**E-F**). Results for the PBPK models (mouse on the left, rat on the right) show simulations (dotted lines) of a 300 mg/kg or 1000 mg/kg oral dose for mouse and rat, respectively, compared with the observed data from the in vivo study reported here (black or blue dots and solid lines; mean ± SD).

**
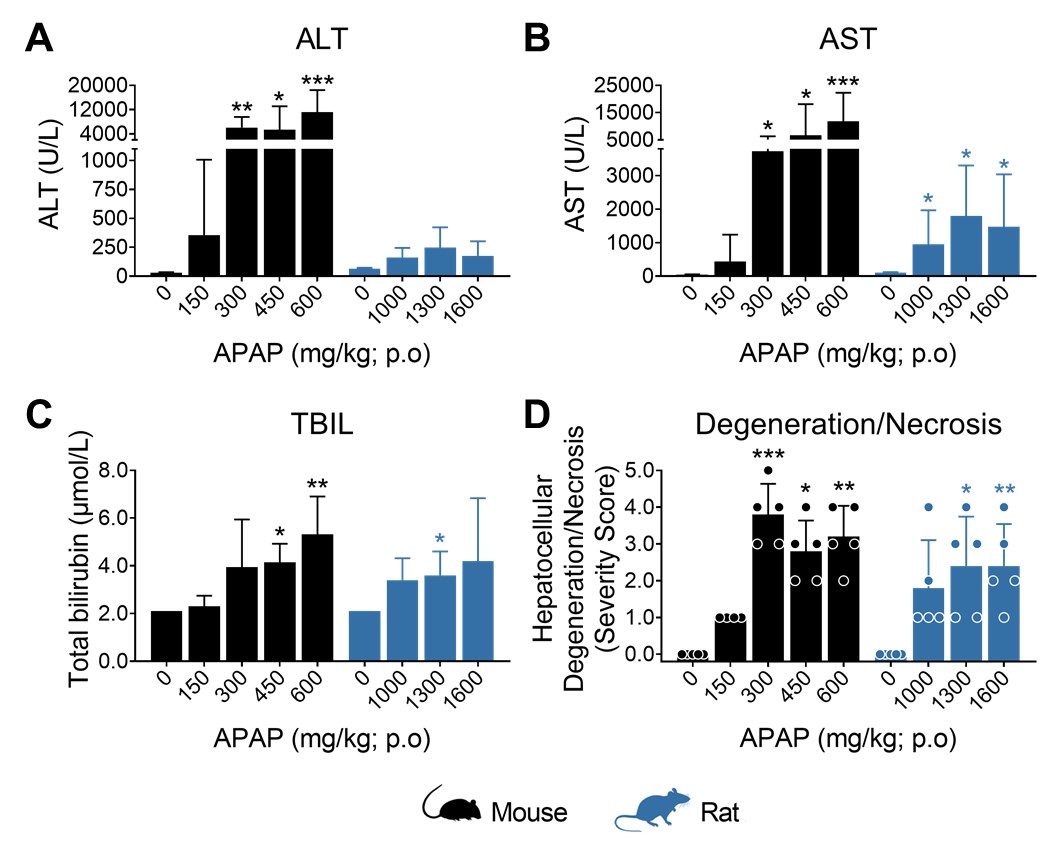
**

**Figure S2.** Dose-ranging study to compare liver tissue responses of mice and rats to APAP. Male C57Bl/6J mice and Sprague-Dawley rats were administered 150-600 mg/kg or 1000-1600 mg/kg APAP, respectively, by oral gavage. We were unable to test higher doses in rat due to the limited solubility of the drug. Liver injury serum markers (ALT (**A**), AST (**B**) and TBIL (**C**)) and degeneration/necrosis (**D**) were evaluated 24 h after dosing. Values are mean ± SD (n=5). In (**D**) severity scores are shown for each animal. Statistical significance was determined between APAP-treated and vehicle control groups per species (Kruskal-Wallis with Dunn’s multiple comparison test). P-values are denoted as *p<0.05, **p<0.01, or ***p<0.001.


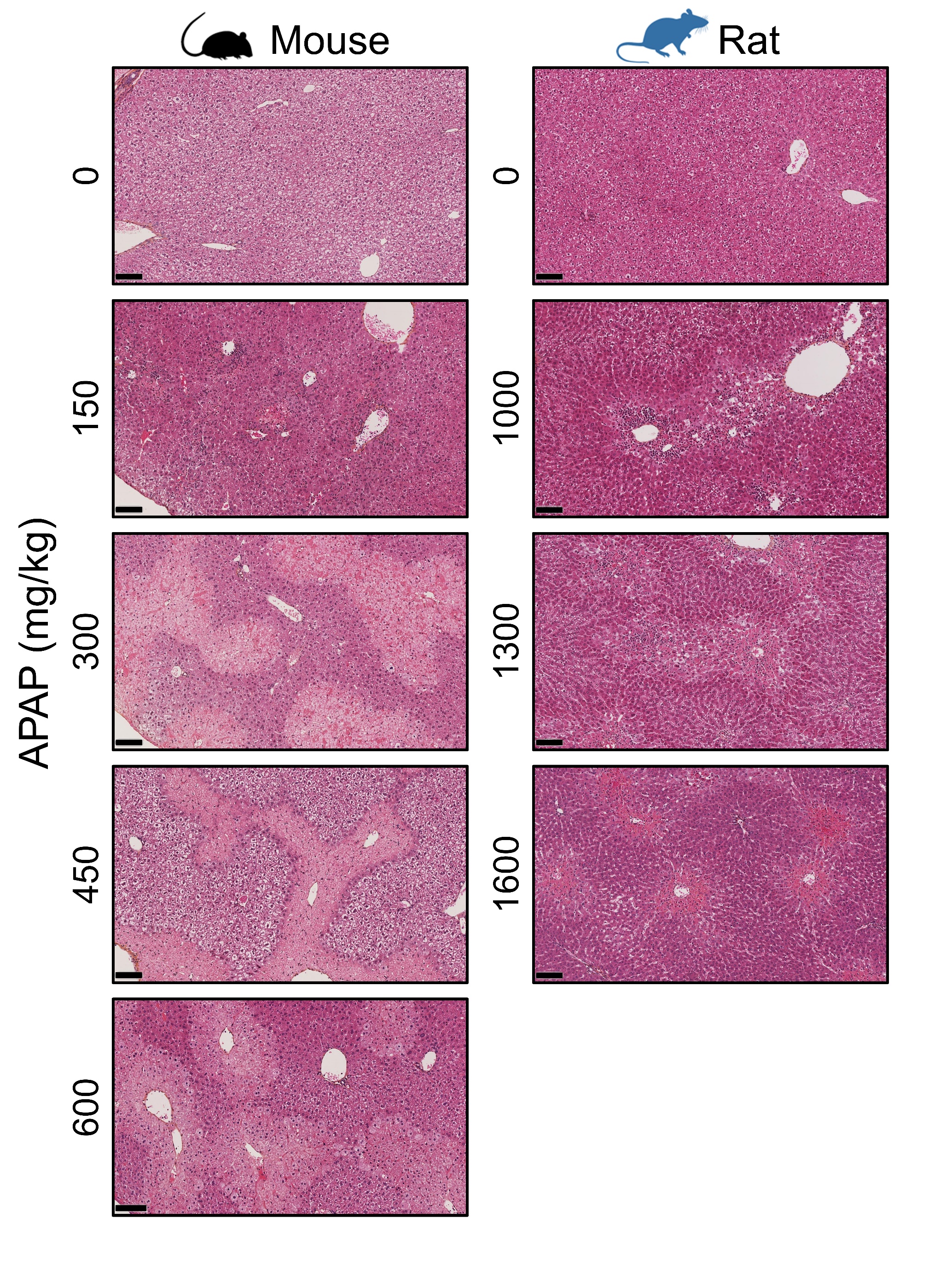


**Figure S3.** Dose-ranging study to compare liver tissue responses of mice and rats to APAP. Male C57Bl/6J mice and Sprague-Dawley rats were administered 150-600 mg/kg or 1000-1600 mg/kg APAP, respectively, by oral gavage. Representative images of hematin-eosin saffron stained liver sections from mice and rats treated with APAP at the indicated doses. Scale bar = 100 μm.


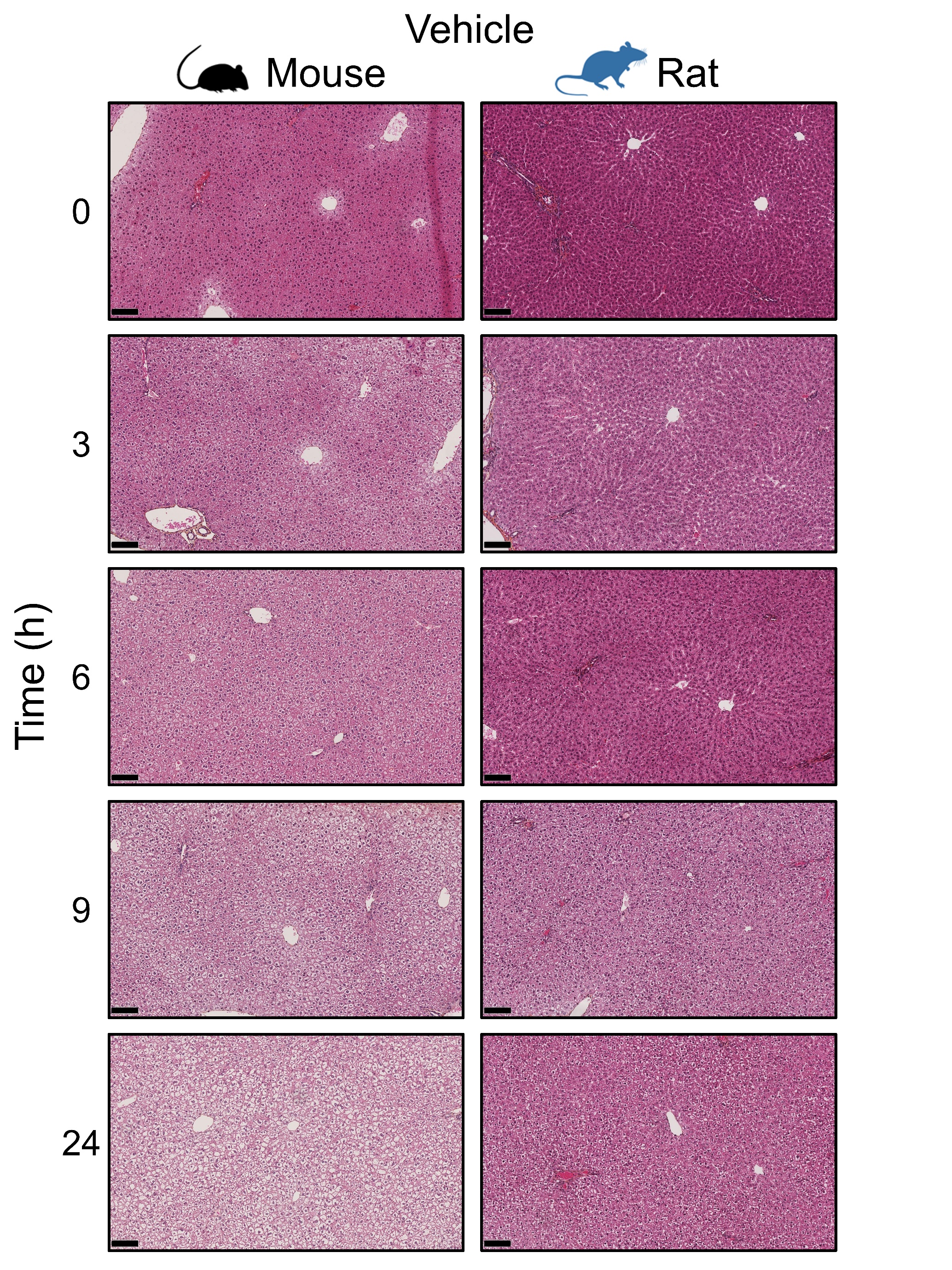


**Figure S4.** Time course study to compare liver tissue responses of mice and rats to APAP. Representative images of hematin-eosin saffron stained liver sections from male C57Bl/6J mice and Sprague-Dawley rats at baseline (0 h) or treated with vehicle (1 % w/v hydroxyethylcellulose) by oral gavage at the indicated time points. Scale bar = 100 μm.


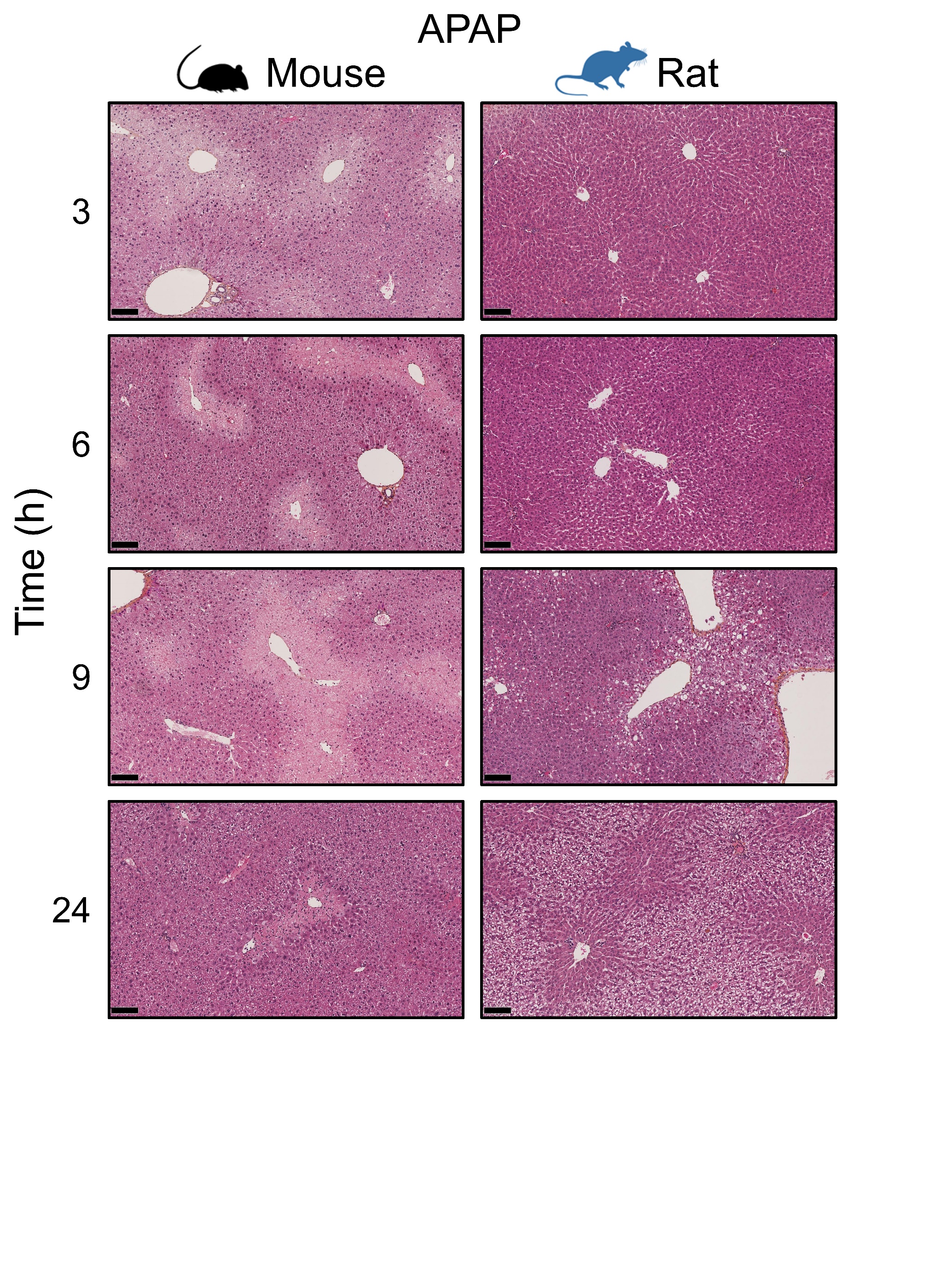


**Figure S5.** Time course study to compare liver tissue responses of mice and rats to APAP. Representative images of hematin-eosin saffron stained liver sections from male C57Bl/6J mice and Sprague-Dawley rats treated with 300 mg/kg and 1000 mg/kg, respectively, by oral gavage at the indicated time points. Scale bar = 100 μm.


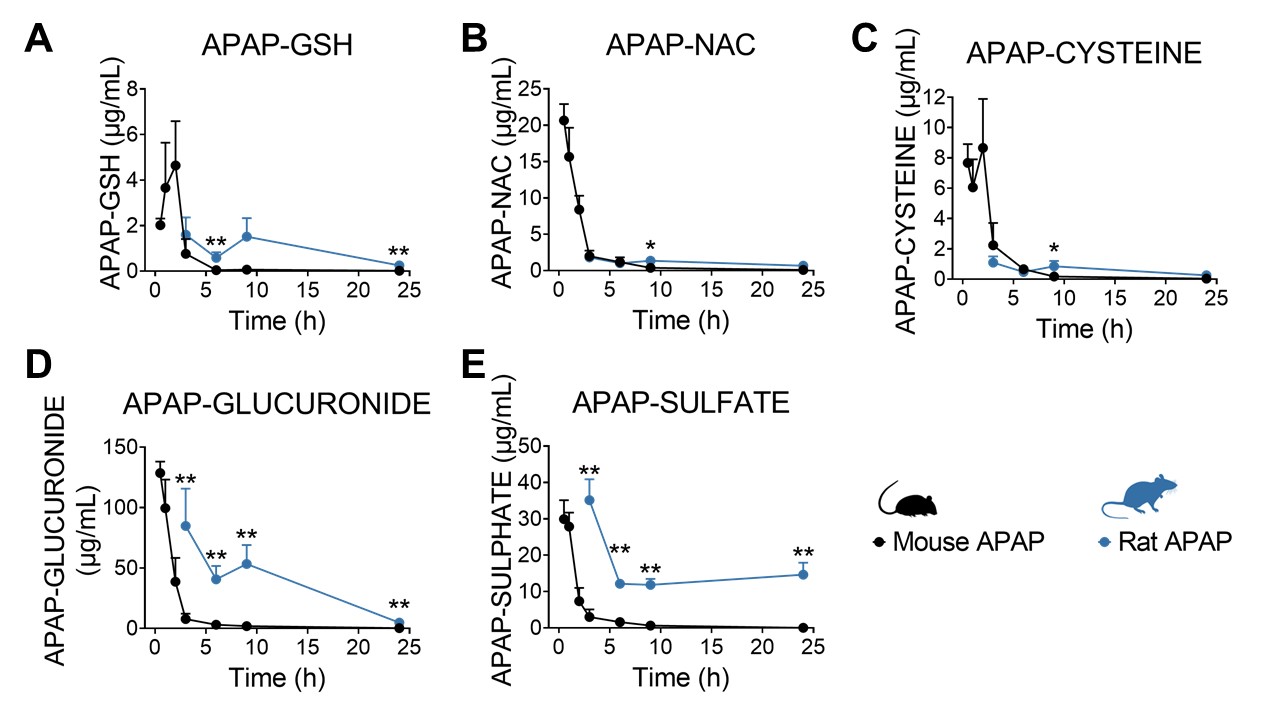


**Figure S6.** Pharmacokinetic quantification of APAP metabolites (APAP-GSH (**A**), APAP-NAC (**B**), APAP-Cysteine (**C**), APAP-Glucuronide (**D**) and APAP-Sulfate (**E**)) in the plasma of C57Bl/6J mice (0.5, 1, 2, 3, 6, 9 and 24 h) and Sprague-Dawley rats (3, 6, 9 and 24 h) exposed to 300 mg/kg and 1000 mg/kg APAP, respectively. Values are mean ± SD (n=5). Statistical significance was determined between APAP-treated mice and rats where possible per time point (Mann-Whitney U test). P-values are denoted as *p<0.05, and **p<0.01.


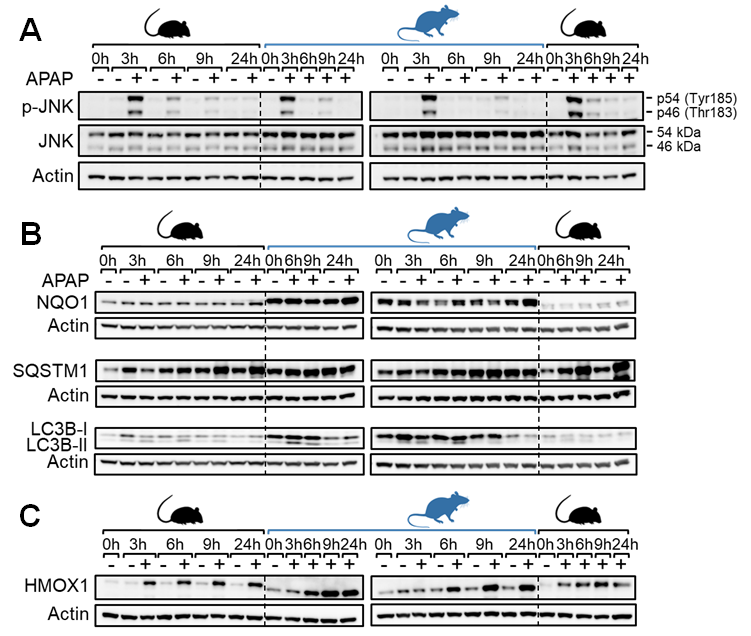


**Figure S7.** Uncut immunoblots showing (**A**) APAP-induced phosphorylation of c-Jun N-terminal kinase (JNK) at Tyr185 (p56) and Thr183 (p46) and protein expression levels of (**B**) NQO1, SQSTM1, LC3B-I and -II, and (**C**) HMOX1. Samples are pooled from 5 animals per time point. Protein levels were normalized to β-actin. To allow for a fair comparison across species when mouse and rat samples had to be run on separated gels due to the number of time points/treatment conditions, few samples from the other species were added to each gel as internal controls, and the two gels were exposed together.


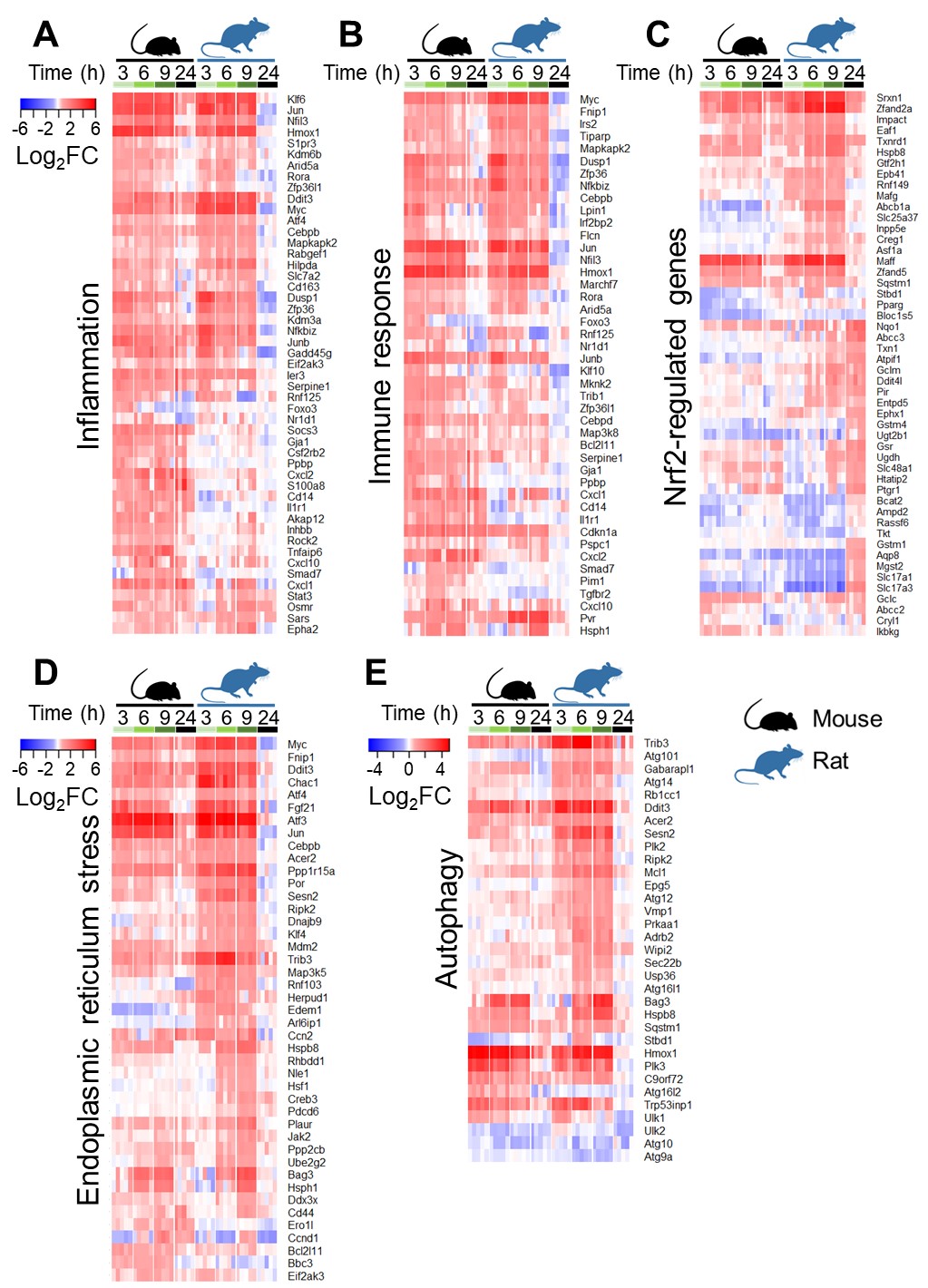


**Figure S8.** Heatmaps showing changes in the expression of genes regulating (**A**) inflammatory, (**B**) immune, (**C**) oxidative stress, (**D**) endoplasmic reticulum (ER) stress responses, and (**E**) autophagy in the livers of C57Bl/6J mice and Sprague Dawley rats treated with 300 mg/kg and 1000 mg/kg APAP, respectively (n=5) at 3, 6, 9, and 24 hours. Data are expressed as log_2_ fold-change *versus* time-matched vehicle control animals.


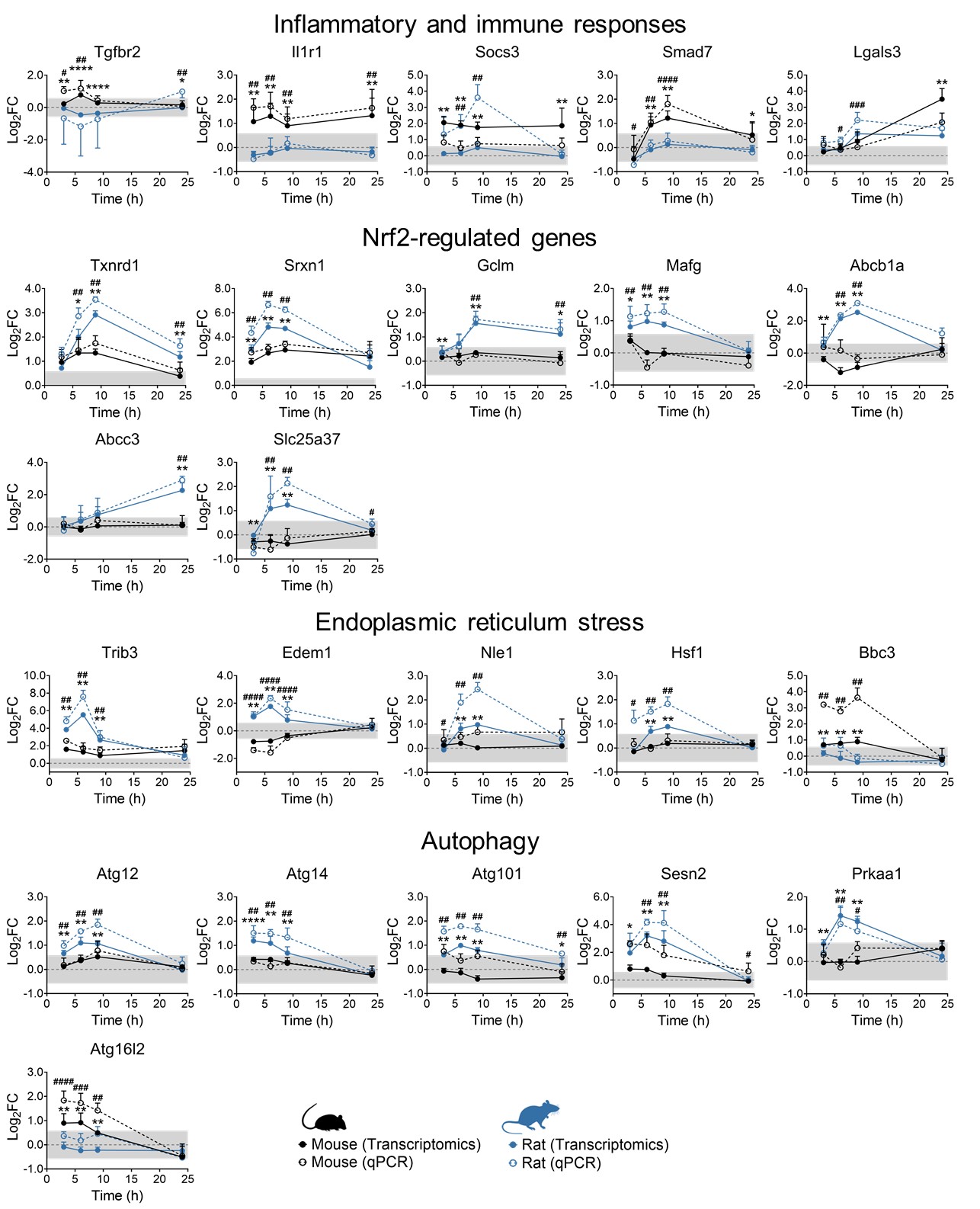


**Figure S9.** mRNA expression levels of representative genes implicated in the indicated processes, as determined by qPCR analysis. Expression levels were normalized to GAPDH. Data are expressed as log_2_ fold-change versus time-matched vehicle control animals. Values are mean ± SD (n=5). Unpaired t-test or Mann-Whitney U test, as appropriate. P-values are denoted as *p<0.05, **p<0.01, or ****p<0.0001, comparison of mouse transcriptomics versus rat transcriptomics, or ^#^p<0.05, ^##^p<0.01, ^###^p<0.001, ^####^p<0.0001, comparison of mouse qPCR versus rat qPCR.


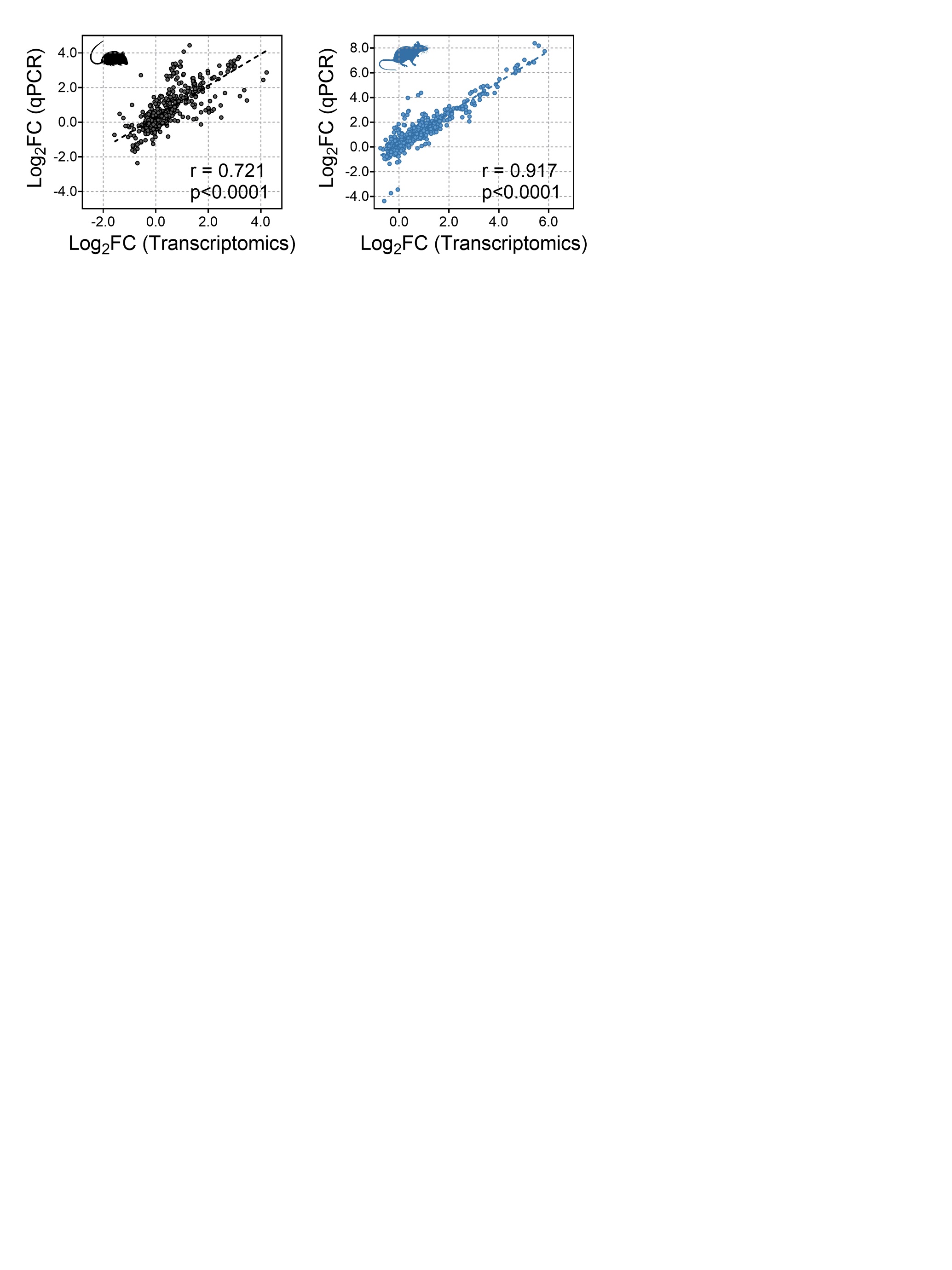


**Figure S10.** Linear correlations between transcriptomics and qPCR data. Values are expressed as log_2_ fold-change versus time-matched vehicle control animals. Pearson correlation coefficient (r) and p value for each species are shown in the plot.


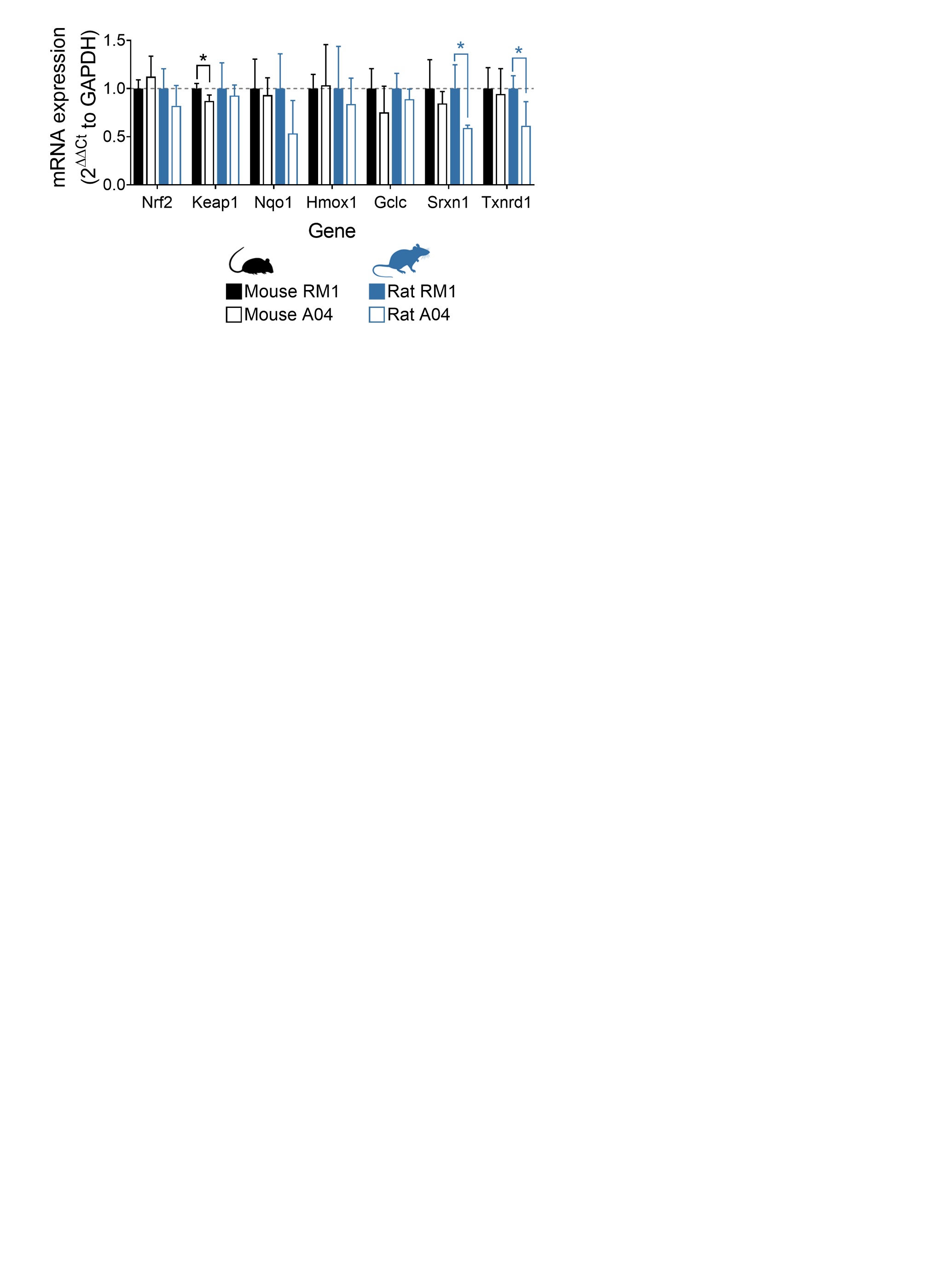


**Figure S11.** mRNA expression levels of representative Nrf2-modulated genes in mice and rat fed with diets containing relatively high (A04, Safe, France) and low (RM1, Special Diet Services, UK) levels of arsenic. Male C57Bl/6J mice and male Sprague-Dawley rats were assigned to either RM1 or A04 diet for 7 days. Expression levels were normalized to GAPDH. Values are mean ± SD (n=4). Statistical significance was determined against RM1-fed animals (Unpaired t-test or Mann-Whitney U test, as appropriate). P-values are denoted as *p<0.05.


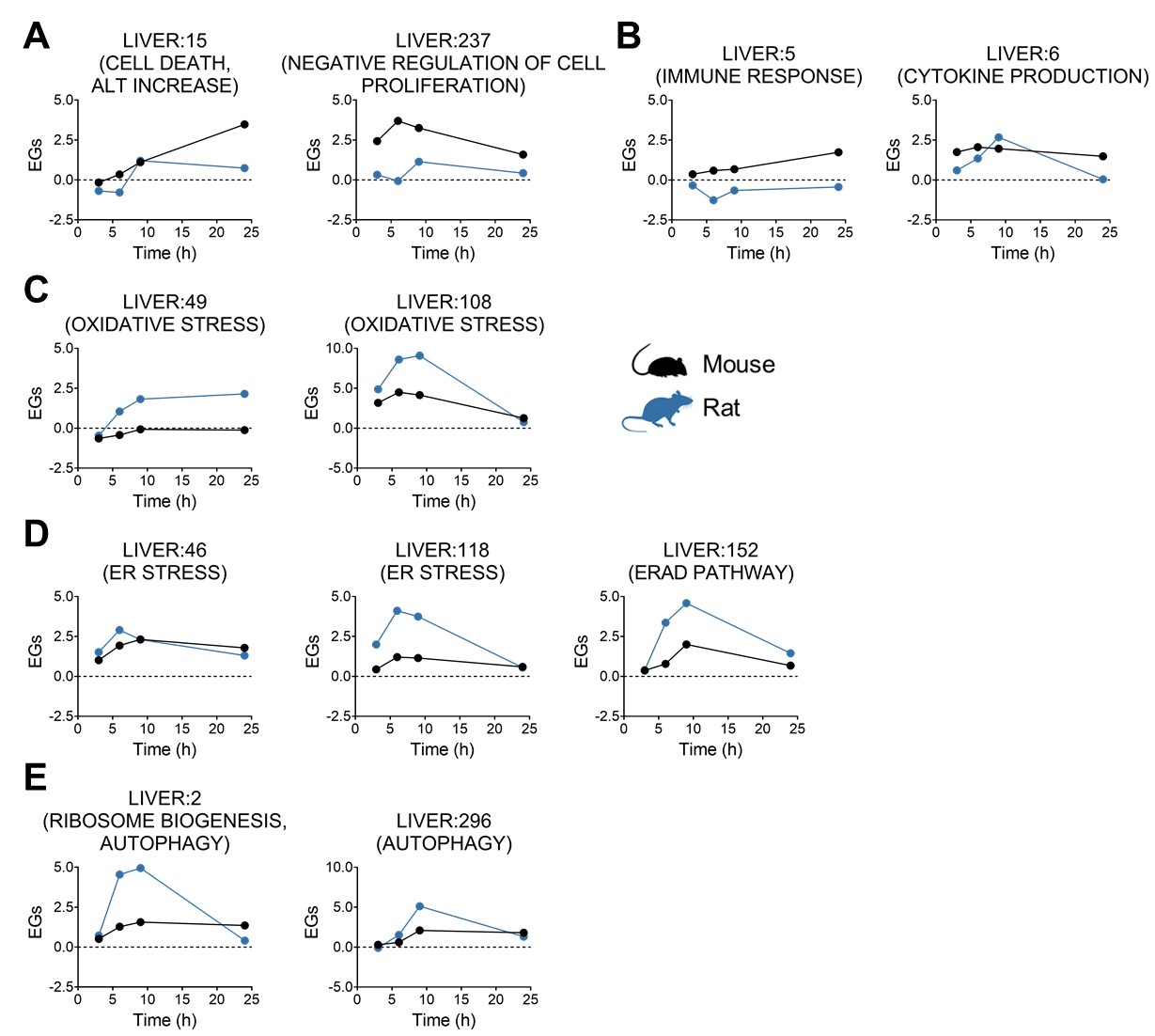


**Figure S12.** Weighted gene co-expression network analysis (WGCNA) of differentially expressed genes in APAP-treated mice and rats using the TXG-MAPr web tool (https://txg-mapr.eu). A numeric Eigengene score (EGs) that aggregates fold-change values for the underlying genes in the module was calculated for both species at each time point. Relevant modules involved in the modulation of (**A**) cell death, (**B**) immune, inflammatory, (**C**) oxidative stress and (**D**) endoplasmic reticulum stress responses, and (**E**) autophagy**.** ALT, alanine aminotransferase; ER, endoplasmic reticulum; ERAD, endoplasmic-reticulum-associated protein degradation. See File S3 for the full list of perturbed modules in both species.


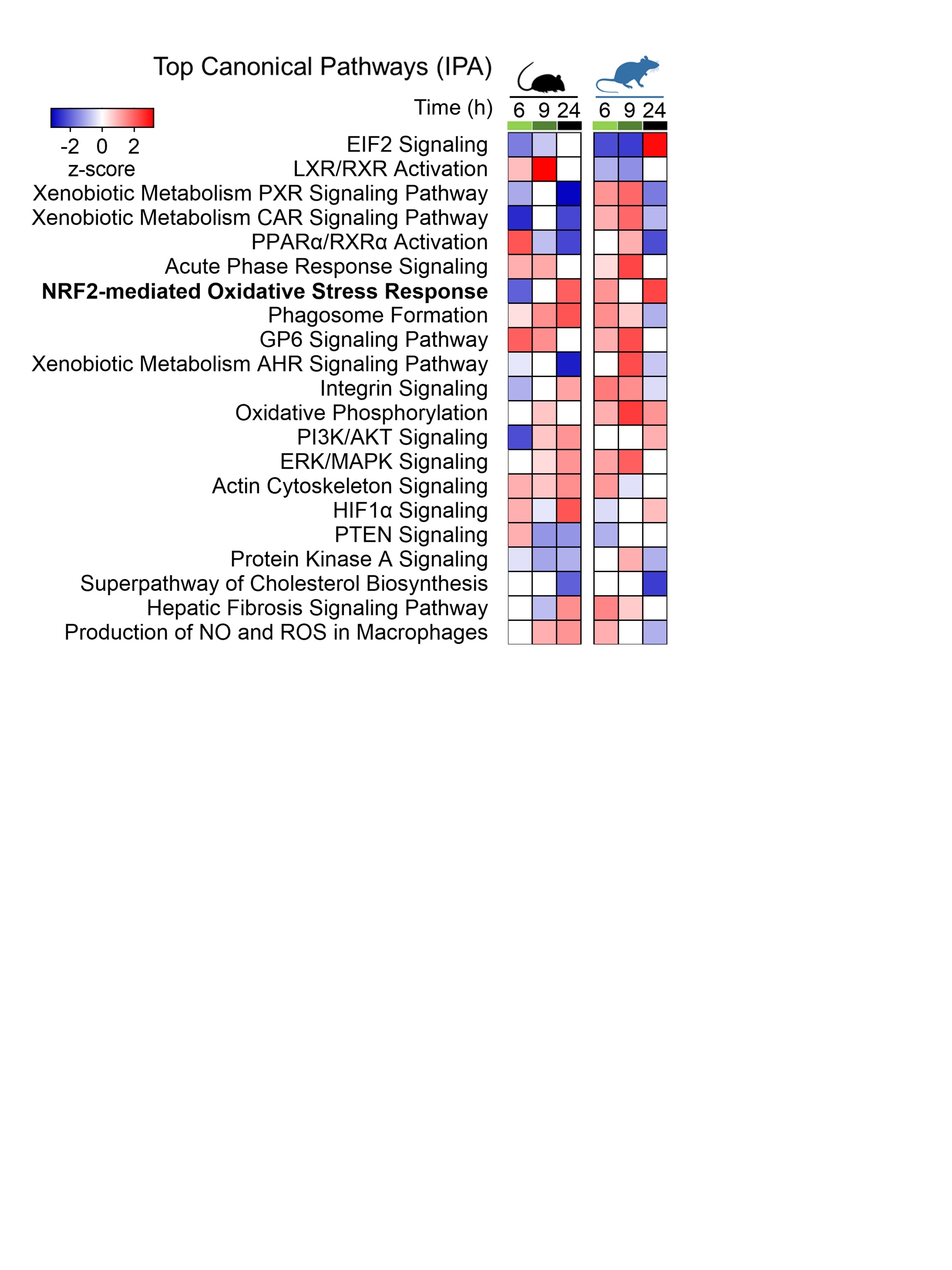


**Figure S13.** Comparative analysis of top canonical pathways (IPA) in the liver of mice and rats treated with acetaminophen (APAP) 300 mg/kg and 1000 mg/kg, respectively (n=5) at 6, 9, and 24 hours (SWATH proteomics). For each function, a z-score was calculated at each time point against time-matched vehicle control animals.

# Supplementary files

**File S1 – Calculation of hepatic NAPQI burden:** Total hepatic NAPQI burden based on glutathione depletion in mice and rats.

**File S2 – GSEA:** Differentially expressed genes (DEGs) and Gene Set Enrichment Analysis (GSEA) in the livers of C57Bl/6J mice and Sprague Dawley rats treated with 300 mg/kg and 1000 mg/kg acetaminophen (APAP), respectively (n=5) at 3, 6, 9, and 24 hours. Selected GO processes shown in the main paper are highlighted in green.

**File S3 – WGCNA:** Weighted gene co-expression network analysis (WGCNA) in the livers of C57Bl/6J mice and Sprague Dawley rats treated with 300 mg/kg and 1000 mg/kg acetaminophen (APAP), respectively (n=5) at 3, 6, 9, and 24 hours. The list of the top 50 most significant genes in each module (ordered by significance) and their relative log_2_ fold-change are also provided.

**File S4 – Binned Protein Expression:** Basal protein expression levels grouped into bins. Log_2_ transformed normalised protein expression values from SWATH proteomics in the livers of untreated (0 h) C57Bl/6J mice and Sprague Dawley rats (n=5) were ranked and grouped into 10 bins. Proteins with the lowest abundance were assigned to bin 1, and those with the highest abundance to bin 10. A bin value of 0 was assigned to proteins that were not detected.

# Supplementary references

Andersen CL, Jensen JL, Orntoft TF. 2004. Normalization of real-time quantitative reverse transcription-pcr data: A model-based variance estimation approach to identify genes suited for normalization, applied to bladder and colon cancer data sets. Cancer Res. 64(15):5245-5250.

Ashburner M, Ball CA, Blake JA, Botstein D, Butler H, Cherry JM, Davis AP, Dolinski K, Dwight SS, Eppig JT et al. 2000. Gene ontology: Tool for the unification of biology. The gene ontology consortium. Nat Genet. 25(1):25-29.

Copple IM, Lister A, Obeng AD, Kitteringham NR, Jenkins RE, Layfield R, Foster BJ, Goldring CE, Park BK. 2010. Physical and functional interaction of sequestosome 1 with keap1 regulates the keap1-nrf2 cell defense pathway. J Biol Chem. 285(22):16782-16788.

Dai M, Wang P, Boyd AD, Kostov G, Athey B, Jones EG, Bunney WE, Myers RM, Speed TP, Akil H et al. 2005. Evolving gene/transcript definitions significantly alter the interpretation of genechip data. Nucleic Acids Res. 33(20):e175.

Demichev V, Messner CB, Vernardis SI, Lilley KS, Ralser M. 2020. Dia-nn: Neural networks and interference correction enable deep proteome coverage in high throughput. Nat Methods. 17(1):41-44.

Gautier L, Cope L, Bolstad BM, Irizarry RA. 2004. Affy--analysis of affymetrix genechip data at the probe level. Bioinformatics. 20(3):307-315.

Ierusalimschy R. 2006. Programming in lua, second edition. Lua.Org.

Jamei M, Dickinson GL, Rostami-Hodjegan A. 2009. A framework for assessing inter-individual variability in pharmacokinetics using virtual human populations and integrating general knowledge of physical chemistry, biology, anatomy, physiology and genetics: A tale of 'bottom-up' vs 'top-down' recognition of covariates. Drug Metab Pharmacokinet. 24(1):53-75.

Jamei M, Marciniak S, Edwards D, Wragg K, Feng K, Barnett A, Rostami-Hodjegan A. 2013. The simcyp population based simulator: Architecture, implementation, and quality assurance. In Silico Pharmacol. 1:9.

Johnson M, Zaretskaya I, Raytselis Y, Merezhuk Y, McGinnis S, Madden TL. 2008. Ncbi blast: A better web interface. Nucleic Acids Res. 36(Web Server issue):W5-9.

Musther H, Harwood MD, Yang J, Turner DB, Rostami-Hodjegan A, Jamei M. 2017. The constraints, construction, and verification of a strain-specific physiologically based pharmacokinetic rat model. J Pharm Sci. 106(9):2826-2838.

Percie du Sert N, Hurst V, Ahluwalia A, Alam S, Avey MT, Baker M, Browne WJ, Clark A, Cuthill IC, Dirnagl U et al. 2020. The arrive guidelines 2.0: Updated guidelines for reporting animal research. PLoS Biol. 18(7):e3000410.

Ritchie ME, Phipson B, Wu D, Hu Y, Law CW, Shi W, Smyth GK. 2015. Limma powers differential expression analyses for rna-sequencing and microarray studies. Nucleic Acids Res. 43(7):e47.

Rowland Yeo K, Jamei M, Yang J, Tucker GT, Rostami-Hodjegan A. 2010. Physiologically based mechanistic modelling to predict complex drug-drug interactions involving simultaneous competitive and time-dependent enzyme inhibition by parent compound and its metabolite in both liver and gut - the effect of diltiazem on the time-course of exposure to triazolam. Eur J Pharm Sci. 39(5):298-309.

Sutherland JJ, Webster YW, Willy JA, Searfoss GH, Goldstein KM, Irizarry AR, Hall DG, Stevens JL. 2018. Toxicogenomic module associations with pathogenesis: A network-based approach to understanding drug toxicity. Pharmacogenomics J. 18(3):377-390.

Vandeputte C, Guizon I, Genestie-Denis I, Vannier B, Lorenzon G. 1994. A microtiter plate assay for total glutathione and glutathione disulfide contents in cultured/isolated cells: Performance study of a new miniaturized protocol. Cell Biol Toxicol. 10(5-6):415-421.

Wang S, García-Seisdedos D, Prakash A, Kundu DJ, Collins A, George N, Fexova S, Moreno P, Papatheodorou I, Jones AR et al. 2022. Integrated view and comparative analysis of baseline protein expression in mouse and rat tissues. PLoS computational biology. 18(6):e1010174.

Wisniewski JR, Hein MY, Cox J, Mann M. 2014. A "proteomic ruler" for protein copy number and concentration estimation without spike-in standards. Mol Cell Proteomics. 13(12):3497-3506.

Yu G, Wang LG, Han Y, He QY. 2012. Clusterprofiler: An r package for comparing biological themes among gene clusters. OMICS. 16(5):284-287.

Zaru R, Orchard S, UniProt C. 2023. Uniprot tools: Blast, align, peptide search, and id mapping. Curr Protoc. 3(3):e697.

Zhu Y, Orre LM, Zhou Tran Y, Mermelekas G, Johansson HJ, Malyutina A, Anders S, Lehtio J. 2020. Deqms: A method for accurate variance estimation in differential protein expression analysis. Mol Cell Proteomics. 19(6):1047-1057.
